# Microglial Therapeutic Potential in Alzheimer’s Disease: From Phenotypic Diversity to Targeted Interventions

**DOI:** 10.3390/ph19101603

**Published:** 2026-10-09

**Authors:** Maria-Raluca Gogu, Ivona Mazarianu, Gabriela-Dumitrita Stanciu, Bogdan-Ionel Tamba, Mitica Ciorpac, Dragos-Lucian Gorgan

**Affiliations:** 1 Department of Biology, Faculty of Biology, Alexandru Ioan Cuza University of Iasi, 700506 Iasi, Romania; raluca.gogu@umfiasi.ro (M.-R.G.); mitica.ciorpac@umfiasi.ro (M.C.);; 2 Advanced Research and Development Center for Experimental Medicine “Prof. Ostin C. Mungiu”, Grigore T. Popa University of Medicine and Pharmacy Iasi, 700454 Iasi, Romania; ivona.mazarianu@umfiasi.ro (I.M.); bogdan.tamba@umfiasi.ro (B.-I.T.); 3 Department of Pharmacology, Clinical Pharmacology and Algesiology, Grigore T. Popa University of Medicine and Pharmacy Iasi, 700115 Iasi, Romania

**Keywords:** Alzheimer’s disease, microglia, neuroinflammation, β-amyloid, tau pathology, disease-associated microglia, microglia-targeted therapies, neurodegeneration

## Abstract

Alzheimer’s disease (AD) is a progressive neurodegenerative disorder characterized by β-amyloid (Aβ) deposition, tau pathology and cognitive decline. Increasing evidence from genetic, transcriptomic, experimental and clinical studies has identified microglia as key regulators of AD pathogenesis. Under physiological conditions, microglia maintain central nervous system homeostasis through immune surveillance, phagocytosis and neurotrophic support. However, persistent exposure to pathological stimuli promotes a shift toward dysfunctional inflammatory states that contribute to neuroinflammation, synaptic impairment, tau propagation and neurodegeneration. Recent advances have revealed substantial microglial heterogeneity, highlighting the existence of dynamic and disease stage-dependent activation states rather than fixed phenotypes. In early disease stages, microglia can promote Aβ clearance and tissue repair, whereas chronic activation may exacerbate pathological processes. These findings have driven the development of microglia-targeted therapeutic strategies aimed at enhancing protective functions while limiting maladaptive inflammatory responses. This review summarizes current knowledge regarding microglial involvement in AD and discusses emerging therapeutic approaches, ranging from immunomodulation and metabolic reprogramming to gene-based and nanotechnology-driven interventions, with a focus on their translational potential.

## 1. Introduction

AD is the most common cause of dementia, accounting for approximately 60–70% of all cases worldwide. It is estimated that around 10% of individuals aged 65 years and older are affected by AD, with the prevalence increasing to 32% among those over 85 years of age [1]. Patients with AD experience a progressive decline in memory and cognitive function, affecting language, visuospatial abilities and executive functions [2,3]. The neuropathological hallmarks of AD include the accumulation of β-amyloid (Aβ) plaques in the extracellular space between neurons and the formation of intraneuronal neurofibrillary tangles composed of hyperphosphorylated tau protein [4,5,6,7,8]. According to the amyloid cascade hypothesis, Aβ accumulation represents the initiating pathological event that triggers a series of downstream molecular and cellular processes, ultimately leading to synaptic dysfunction, neuronal degeneration, neuroinflammation and cognitive decline [9,10,11]. Neuroinflammation is a complex cascade of inflammatory processes within the central nervous system (CNS) characterized by the sustained release of pro- and anti-inflammatory cytokines, chemokines, reactive oxygen species (ROS) and other signaling mediators by resident microglia [12]. Microglia are the brain’s resident innate immune cells and primary immune sentinels of the human CNS [13], arising from yolk sac progenitors [14] and derived from early embryonic myeloid-monocytic cells [15]. Under normal physiological conditions, microglia are capable of self-renewal throughout the entire lifespan of the organism [16]. They are estimated to comprise around 10% of all cells in the adult central nervous system [17]. Microglia continuously monitor the central nervous system throughout the lifetime, shaping key processes such as host defense, homeostasis, wound healing and immune response regulation [17], production of cytokine, chemokine and neurotrophic factors, protein aggregation [18], release of soluble factors, phagocytosis and the refinement of neural circuits [14]. In response to various stressors, they can migrate and undergo structural and functional changes. As the brain’s primary immunocompetent cells, microglia play key roles in both neuroprotection and neurodegeneration [19]. They are generally beneficial, promoting neuronal survival by secreting growth factors and anti-inflammatory cytokines such as transforming growth factor beta (TGF-β), interleukin-1 receptor antagonist (IL-1Ra) and interleukin-10 (IL-10) [20]. But tissue injury and neurodegeneration can drive microglia toward a pro-inflammatory phagocytic phenotype, characterized by the release of a broad spectrum of inflammatory mediators, including cytokines such as tumor necrosis factor α (TNF-α) and several interleukins (IL-1β, IL-6, IL-12, IL-17, IL-18, and IL-23), chemokines including chemokine ligand 12 (CCL12) and C-X-C motif chemokine ligand 10 (CXCL10), as well as ROS and reactive nitrogen species (RNS) [21,22,23]. Glial reactivity in AD is better understood as a dynamic continuum than as a set of fixed binary categories. The classic microglial (M1/M2) phenotypes remain useful shorthand, but sources emphasize that microglia occupy overlapping, context-dependent states that vary with tissue niche, disease stage and local inflammatory cues, and that intermediate or mixed phenotypes are common rather than exceptional [24,25,26]. For microglia, this continuum begins with an early, often protective response to pathology, when cells sense damage, proliferate, migrate toward amyloid or tau lesions, and attempt to contain or clear toxic material [12]. In several AD models, early-response microglia upregulate cell-cycle and chemokine programs, whereas later-response cells shift toward immune-response, interferon and MHC-related signatures, reflecting a transition from surveillance and containment to a more sustained inflammatory state [24,26,27]. This stage dependence is also visible in disease-associated microglia programs. Disease-associated microglia (DAM) and other related states are enriched around plaques, express genes involved in phagocytosis and are often interpreted as adaptive in early phases but potentially maladaptive when activation becomes chronic and excessive [28,29]. Taken together, these findings support a stage-dependent microglial model in which early responses are typically aimed at sensing injury and restricting damage, intermediate phases amplify phagocytosis, cytokine signaling and tissue remodeling, and late phases can either resolve pathology or lock the tissue into chronic maladaptive inflammation, depending on the local pathology and the duration of stimulation [30,31,32].

Accordingly, microglia-targeted therapies seek to restore microglial homeostasis through selective phenotypic modulation, preserving neuroprotective functions, including Aβ clearance and tissue surveillance, while attenuating maladaptive inflammatory cascades that contribute to neurodegeneration. The expanding range of therapeutic targets, encompassing immune receptors, purinergic and inflammatory signaling pathways, complement components, nuclear receptors, epigenetic mechanisms and gene-based interventions, reflects both the considerable potential and the inherent complexity of microglia-centered therapies in AD. While drug development in AD has traditionally focused on reducing Aβ and tau pathology, increasing attention has been directed toward the contribution of microglia to disease pathogenesis. This shift has been largely driven by strong genetic evidence linking microglial pathways to AD risk and progression. Consequently, microglia have emerged both as key mediators of disease mechanisms and as promising therapeutic targets. This review provides a comprehensive overview of microglia-targeted therapeutic strategies in AD, summarizing current evidence from cell-based experiments, preclinical animal studies and clinical investigations, while highlighting emerging microglia-centered approaches with translational potential.

### Literature Search Strategy and Selection Criteria

To establish a structured narrative overview of microglial biology, phenotypic transitions, and targeted interventions in Alzheimer’s disease (AD), a comprehensive literature retrieval was conducted across PubMed/MEDLINE, Web of Science (Core Collection), and Scopus, supplemented by clinical trial registries (ClinicalTrials.gov), covering peer-reviewed articles published up to 2026. The search was performed between 15 May 2026 and 15 August 2026, covering all peer-reviewed articles indexed from database inception up to 15 August 2026. The search strategy employed combinations of Medical Subject Headings terms and Boolean operators: (“microglia” OR “microglial activation” OR “microglial heterogeneity” OR “disease-associated micro-glia” OR “DAM” OR “microglial neurodegenerative phenotype” OR “MGnD” OR “dark microglia” OR “interferon-response microglia”) AND (“Alzheimer’s disease” OR “amyloid-beta” OR “tauopathy” OR “neuroinflammation” OR “synaptic pruning”) AND (“therapeutic targeting” OR “immunomodulation” OR “TREM2” OR “APOE” OR “NLRP3 inflammasome” OR “CSF1R” OR “PPAR agonists” OR “cannabinoids” OR “mesenchymal stem cells” OR “gene therapy” OR “nanoparticles” OR “clinical trial”). Literature retrieval, eligibility screening, and contextual data appraisal were performed collaboratively by the lead authors (M.-R.G. and I.M.) with conceptual oversight from senior co-authors (G.-D.S., B.-I.T., M.C. and D.-L.G.).

Studies were evaluated according to predefined eligibility criteria:

Inclusion criteria—(1) primary experimental investigations in rodent models and human cell-derived platforms (e.g., hiPSC-microglia); (2) high-resolution single-cell and single-nucleus RNA sequencing (snRNA-seq) or spatial transcriptomic studies in post-mortem human AD brain specimens; (3) completed or active Phase I–III randomized controlled clinical trials evaluating neuroinflammatory and microglia-modulating candidates; and (4) seminal reviews selectively included to provide contextual framework.

Exclusion criteria—Studies without formal peer review, non-English publications, conference abstracts lacking complete methodology, and investigations focusing exclusively on peripheral immunosuppressive therapies without assessing central neuroimmune parameters.

## 2. Microglial Heterogeneity and Dynamic Responses in Alzheimer’s Disease

### 2.1. Microglial Phenotypes

**Figure 1:**
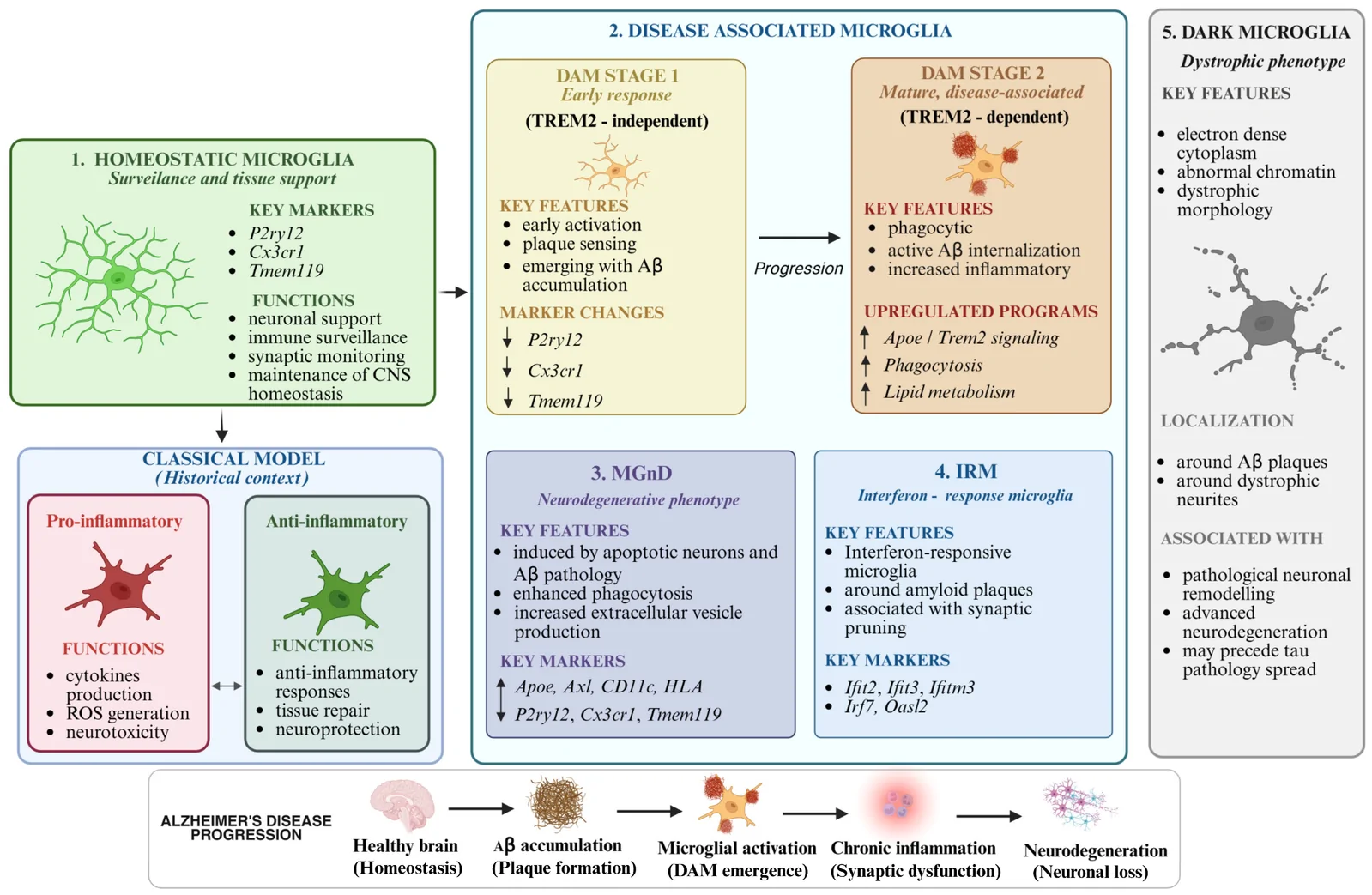
Microglial phenotypic transition during AD progression. Microglia exist in a homeostatic state characterized by expression of *P2ry12*, *Cx3cr1* and *Tmem119* and functions including neuronal support, immune surveillance, and synaptic monitoring. The classical binary pro-/anti-inflammatory framework is included strictly for historical context, having been superseded by in vivo transcriptomic states. During AD, microglia undergo disease-specific transitions, including DAM, which develop through a TREM2-independent early DAM (stage 1) and a TREM2-dependent mature DAM (stage 2), accompanied by progressive loss of homeostatic markers and increased phagocytic and inflammatory activity. Other disease-related phenotypes include MGnD, induced by apoptotic neurons and Aβ and IRM, associated with interferon signaling, plaque accumulation and synaptic pruning. In late-stage disease, DAM emerge around Aβ plaques and dystrophic neurons and are linked to pathological neuronal remodeling and neurodegeneration. Black arrows indicate transitions or progression between microglial states and stages of AD pathology, whereas the bidirectional arrow in the classical model indicates phenotypic plasticity between pro- and anti-inflammatory states. Upward and downward arrows indicate increased/upregulated and decreased/downregulated expression or activity, respectively. Colors are used to distinguish the different microglial phenotypes and conceptual categories represented in the figure and do not indicate a quantitative scale. Abbreviations: Aβ, amyloid-β; *Apoe*, apolipoprotein E; *Axl*, AXL receptor tyrosine kinase; CD11c, cluster of differentiation 11c; CNS, central nervous system; *Cx3cr1*, C-X3-C motif chemokine receptor 1; DAM, disease-associated microglia; HLA, human leukocyte antigen; *Ifit2*, interferon-induced protein with tetratricopeptide repeats 2; *Ifit3*, interferon-induced protein with tetratricopeptide repeats 3; *Ifitm3*, interferon-induced transmembrane protein 3; *Irf7*, interferon regulatory factor 7; IRM, interferon-response microglia; MGnD, microglial neurodegenerative phenotype; *Oasl2*, 2′-5′-oligoadenylate synthetase-like 2; *P2ry12*, purinergic receptor P2Y12; ROS, reactive oxygen species; *Tmem119*, transmembrane protein 119; *Trem2*, triggering receptor expressed on myeloid cells 2. Created in BioRender. Mazarianu, I. (2026) https://BioRender.com/fv90n4e (accessed on 25 August 2026).

Homeostatic microglia—under normal physiological conditions, microglia exhibit a relatively quiescent phenotype specialized for monitoring neuronal homeostasis and providing immune surveillance within the neural microenvironment [33]. In the healthy adult brain, these cells predominantly display a ramified morphology with extensively branched, highly dynamic processes that constantly extend and retract, enabling them to sense and respond to subtle alterations in their surroundings (Figure 1) [34].

Despite being traditionally regarded as a functionally homogeneous population, human adult microglia display considerable intrinsic heterogeneity. At baseline, homeostatic microglia are sustained by a distinct gene-expression profile enriched in core regulators, notably *TMEM119*, *P2RY12*, and *CX3CR1* [35]. Morphometric mapping of post-mortem human brain specimens has demonstrated that microglial densities are largely preserved across neocortical areas (including frontal and occipital lobes) and the hippocampus, with modest regional elevation in the substantia nigra [36,37]. Microglial specialization is governed by intrinsic responses to distinct regional microenvironments rather than gross fluctuations in cell numbers. This structural heterogeneity establishes territory-dependent surveillance setpoints, directly contributing to the differential susceptibility and localized immune reactivity observed across vulnerable CNS regions in neurodegenerative conditions. From a preclinical perspective, rodent studies have demonstrated substantial regional variation in microglial distribution. In mice, microglia account for approximately 5% of cells in the cerebral cortex and cerebellum, compared with nearly 12% in the substantia nigra, and are more abundant in gray than in white matter. Their density generally decreases from the forebrain to the hindbrain, following a pattern that may reflect CNS developmental organization [38]. With advancing age, resident microglia experience a steady loss of their core homeostatic identity, a transition frequently framed as immune priming or senescence. Key surveillance factors such as *P2RY12* and *TMEM119* are steadily suppressed, accompanied by cytosolic lipid-droplet buildup [39], stress-pathway activation, and dystrophic nuclear reorganization [40,41]. Functionally, these senescent perturbations impede arbor motility and clearance of cellular debris, effectively priming the microglia to mount unconstrained, hyper-reactive inflammatory responses upon secondary pathological challenge [42]. Across the progression of AD pathology, the breakdown of physiological microglial surveillance correlates tightly with the local density of misfolded protein aggregates. In asymptomatic or nascent phases, microglial cells retain sufficient baseline transcriptional machinery to support physiological synaptic remodeling and mediate the targeted uptake of soluble amyloid assemblies [43]. As toxic oligomers condense into fibrillar plaque cores, canonical homeostatic circuits are actively shut down. This intracellular rewiring dismantles standard surveillance routines, steering cells into reactive, lesion-anchored transcriptional states. Under persistent pathological stress, these activated subsets ultimately degrade into exhausted, functionally uncoupled, or fragmented dystrophic phenotypes that fail to sustain tissue protection [42,44]. Under physiological conditions, murine homeostatic programs are characterized by a conserved transcriptional signature comprising *P2ry12*, *Tmem119*, *Siglech*, *Gpr34*, *Socs3*, *Hexb*, *Olfml3*, and *Fcrls* [32]. Some of these core signature genes are conserved across species and maintain surveillance-associated programs in human microglia as well, most notably P2RY12, TMEM119 and CX3CR1 [45].

### 2.2. The Historical M1/M2 Dichotomy

Initial neuroinflammatory frameworks adapted the dichotomous M1/M2 concept from peripheral macrophage assays to differentiate tissue-damaging inflammatory signals from neuroprotective, restorative programs [26]. High-resolution transcriptomic profiling of post-mortem human brain specimens has revealed that this dualistic scheme does not capture microglial phenotypes in vivo [46]. Foundational rodent markers display minimal or undetected expression within the human adult brain parenchyma. Microglia in the diseased CNS simultaneously activate overlapping cytotoxic, resolving, and lipid-clearing programs, making rigid, mutually exclusive categories biologically invalid [47]. These findings suggest that the traditional M1/M2 classification, primarily derived from in vitro studies and peripheral macrophages, oversimplifies the complexity of microglial responses in CNS neurodegeneration and fails to account for additional states such as proliferating microglia [48,49]. Evidence suggests that microglial phenotypes in AD are highly diverse and dynamic, encompassing a continuum of transcriptional states that can coexist and transition in response to the local microenvironment, rather than representing a linear progression between clearly defined phenotypes [26].

### 2.3. Transcriptomic Microglial States

The pronounced heterogeneity of microglia allows these cells to adopt context-dependent responses to pathological stimuli, including infection, tissue injury, and neurodegeneration, thereby contributing to the adaptive plasticity of the CNS. Advances in multi-omics approaches have further enabled the characterization of discrete microglial phenotypes and functional states with unprecedented resolution [50].

Single-cell transcriptomic studies in transgenic rodent models originally identified disease-associated microglia (DAM) [43]. In 5xFAD mice, homeostatic microglia gradually transition into DAM through a two-step activation process consisting of an initial triggering receptor expressed on myeloid cells 2 (TREM2)-independent early DAM (stage 1 DAM), which emerges alongside the progressive accumulation of Aβ plaques, followed by a TREM2-dependent mature DAM (stage 2 DAM). As Aβ and neurofibrillary tangle (NFT) deposition further increase, stage 1 DAM progressively convert into fully activated stage 2 DAM, characterized by a highly phagocytic phenotype and active internalization of Aβ plaques through the upregulation of genes involved in phagocytosis and lipid metabolism [29,43]. This transition is accompanied by the downregulation of homeostatic markers such as *P2ry12*, *Cx3cr1*, and *Tmem119* in early-stage DAM, and the upregulation of genes associated with AD risk and microglial activation, including *Apoe*, *Tyrobp*, *Trem2*, *Cst7*, and *Lpl*, in stage 2 DAM, typically observed during advanced stages of AD (Figure 1) [43]. DAM have been predominantly identified in close proximity to Aβ plaques in both 5xFAD mouse models, where they are involved in inflammatory signaling, phagocytosis, endocytosis, and lipid metabolism, suggesting a role in amyloid clearance [25,43]. In contrast to transgenic mouse models, human post-mortem profiling via snRNA-seq has not confirmed a discrete DAM transcriptional state, detecting mere sporadic upregulation of select myeloid markers such as APOE [51].

Another microglial activation state closely related to DAM is the microglial neuro-degenerative phenotype (MGnD), which has been described across multiple neuro-degenerative disorders, including AD, amyotrophic lateral sclerosis and multiple sclerosis [28]. MGnD polarization is induced in response to the phagocytosis of apoptotic neurons and Aβ-associated pathology through TREM2- and apolipoprotein E (ApoE)-dependent signaling pathways [32,52]. This phenotype is characterized by enhanced phagocytic activity, increased extracellular vesicle production and upregulation of inflammatory and neurodegeneration-associated genes, including *Apoe*, *Axl*, *Clec7a*, *Itgax* (CD11c) and *Hla*, accompanied by the downregulation of homeostatic microglial markers such as *P2ry12*, *Cx3cr1* and *Tmem119*, as well as key transcriptional regulators including *Mafb*, *Mef2a* and *Sall1* (Figure 1) [28,32,53,54]. Comparative transcriptomic analyses have demonstrated substantial overlap between MGnD and DAM gene signatures in both murine models [55] and human AD brains [56], supporting the involvement of this phenotype in neurodegenerative processes.

Activated response microglia (ARM) represent another microglial phenotype identified in both aging and neurodegenerative conditions, such as AD [28]. Transcriptomic studies in a mouse model of AD that expressed mouse APP with three AD-associated mutations (Swedish mutations in exon 16, and Beyreuther/Iberian and Arctic mutations in exon 17) showed that ARMs form a heterogeneous population of reactive microglia with partial overlap with DAM, while maintaining distinct molecular features [57]. ARMs are characterized by increased expression of major histocompatibility complex class II (MHC-II) molecules, tissue repair-related genes such as *Dkk2*, *Gpnmb* and *Spp1*, and AD-associated risk genes including *Apoe* and *Trem2* [58]. These cells are often found surrounding amyloid plaques and amyloid accumulation appears to promote the transition of homeostatic microglia toward the ARM phenotype (Figure 1) [58,59]. Interestingly, ARMs have also been detected in healthy aging brains, including young wild-type mice, suggesting that this phenotype is not exclusively linked to disease but may also reflect age-related microglial adaptations [28].

Another identified microglial phenotype is dark microglia (DM), also known as dystrophic microglia, which have been associated with pathological neuronal remodeling during neurodegeneration [27]. These cells are identified by transmission electron microscopy (TEM) due to their characteristic electron-dense “dark” appearance and abnormal chromatin structure [60,61]. In the APP/PS1 mouse model of AD, dark microglia are significantly increased and are mainly localized around Aβ plaques and dystrophic neurites (Figure 1) [62]. Importantly, this phenotype may precede the spread of tau pathology, suggesting a role in the early stages of neurodegeneration [42].

Interferon-response microglia (IRM) represent a distinct microglial subtype characterized by elevated expression of type I interferon-stimulated genes, including *Ifit2*, *Ifit3*, *Ifitm3*, *Irf7*, and *Oasl2* [63]. Initially identified in AppNL-G-F AD mouse models [64], IRMs have subsequently been detected in 5XFAD AD mouse models and in human AD brains, with their abundance increasing in an age-dependent manner [65]. IRMs are frequently localized around amyloid plaques and have been associated with enhanced amyloid pathology, potentially through IFITM3-mediated modulation of γ-secretase activity (Figure 1) [66]. In addition, interferon-activated microglia contribute to excessive synaptic pruning and dendritic spine loss in C9orf72^−/−^ ALS/FTD mouse models, thereby promoting synaptic dysfunction and cognitive decline in neurodegenerative diseases [67].

Human genetic and transcriptomic evidence provides an important framework for interpreting microglial findings obtained in AD mouse models. AD risk variants are enriched within regulatory regions active in microglia, supporting a contribution of microglial pathways to disease susceptibility; however, disease-associated transcriptional changes in human microglia are not necessarily enriched for genetic risk variants and may therefore also reflect secondary responses to established pathology [68]. Cross-species single-cell analyses further indicate that murine DAM are not fully reproduced in human AD. Human microglia xenografted into AppNL-G-F mice showed only partial overlap with the murine DAM signature and lacked the canonical DAM1/DAM2 transition, while additionally developing antigen-presenting and cytokine-response states that are poorly represented in conventional mouse amyloid models [69,70]. Nevertheless, some pathways appear more conserved; notably, TREM2 contributes to disease-associated state transitions in both murine and human microglia, although the associated transcriptional programs are not identical [69,70]. Human post-mortem studies similarly reveal only partial correspondence with murine DAM/MGnD signatures and identify disease-stage-associated transcriptional changes that may differ in direction from those observed experimentally in mice [68]. Spatial transcriptomic analysis of human AD cortex further demonstrates region- and cortical-layer-dependent microglial alterations, emphasizing that cellular responses are shaped by their anatomical context [71]. Together, these findings indicate that mouse models remain valuable for mechanistic studies, but microglial states and therapeutic responses identified in these models should be considered preclinical proof-of-concept and validated in human genetic, transcriptomic and neuropathological datasets before clinical extrapolation.

## 3. Microglial Mechanisms in Alzheimer’s Disease Pathogenesis

### 3.1. Interactions with Aβ and Tau Pathology

**Figure 2:**
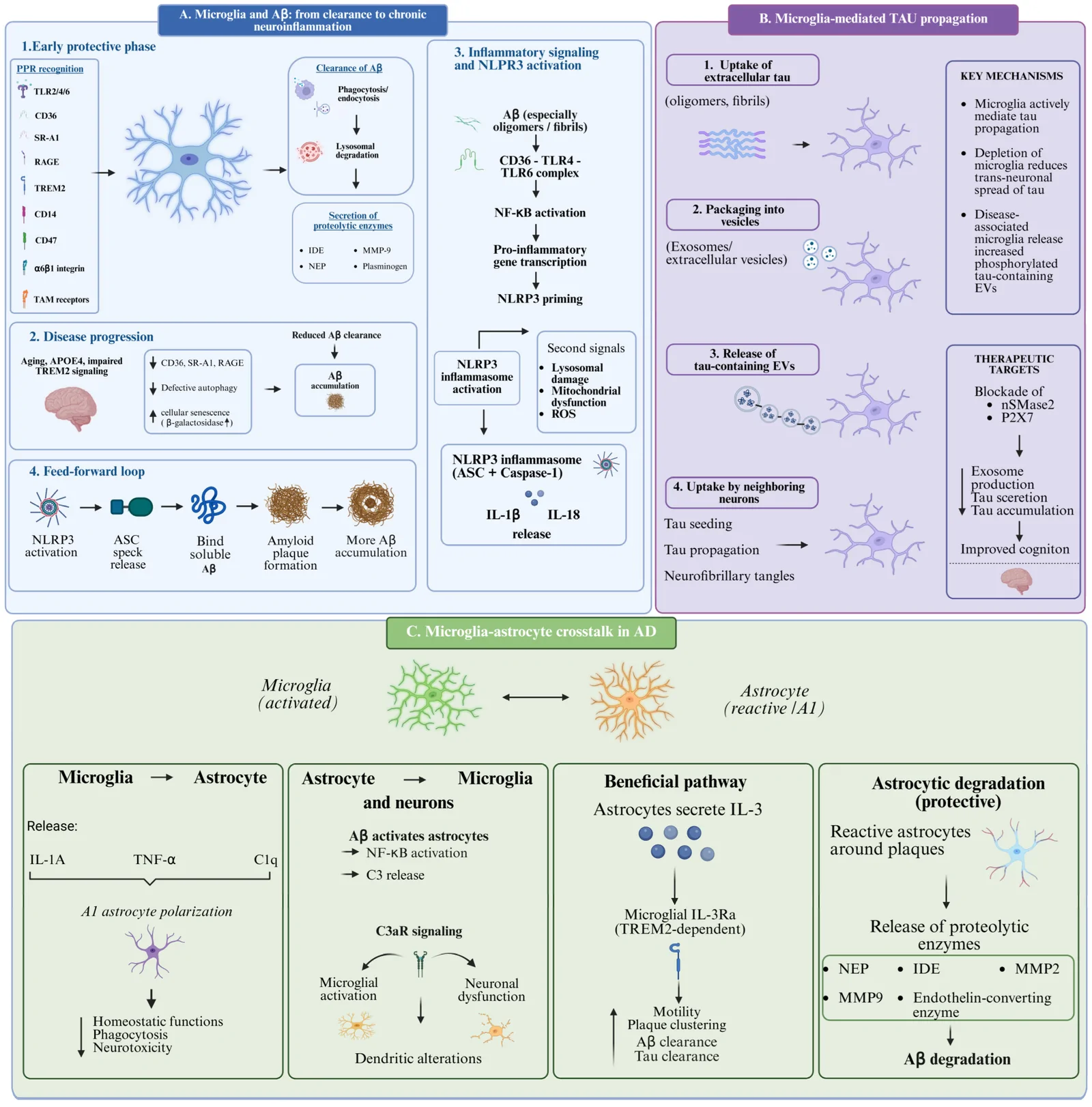
Multifaceted role of microglia in AD pathology. (**A**) The transition of microglia from early protective functions, including Aβ recognition and clearance, to chronic inflammatory activation mediated by the NLRP3 inflammasome, resulting in the release of pro-inflammatory cytokines and enhanced Aβ accumulation. (**B**) Microglia-mediated tau propagation, where extracellular tau is internalized, packaged into extracellular vesicles, and transferred to nearby neurons, promoting tau seeding and neurofibrillary tangle formation. (**C**) The bidirectional crosstalk between activated microglia and reactive astrocytes, which amplifies neuroinflammation, disrupts neuronal function and influences Aβ clearance. These processes contribute to the progression of AD from amyloid pathology and neuroinflammation to tau spread and neurodegeneration. Abbreviations: Aβ, beta-amyloid; AD, Alzheimer’s disease; APOE4, apolipoprotein E ε4 allele; ASC, apoptosis-associated speck-like protein containing a CARD; C1q, complement component 1q; C3, complement component 3; C3aR, complement component 3a receptor; CD14, cluster of differentiation 14; CD36, cluster of differentiation 36; CD47, cluster of differentiation 47; EVs, extracellular vesicles; IDE, insulin-degrading enzyme; IL-1A, interleukin-1 alpha; IL-1β, interleukin-1 beta; IL-3, interleukin-3; IL-18, interleukin-18; IL-3Rα, interleukin-3 receptor alpha; MMP-2, matrix metalloproteinase-2; MMP-9, matrix metalloproteinase-9; NF-κB, nuclear factor kappa B; NEP, neprilysin; NLRP3, NOD-, LRR-, and pyrin domain-containing protein 3; nSMase2, neutral sphingomyelinase 2; P2X7, purinergic receptor P2X7; PRRs, pattern recognition receptors; RAGE, receptor for advanced glycation end products; SRA-1, scavenger receptor class A member 1; TAU, microtubule-associated protein tau; TLR4, toll-like receptor 4; TLR6, toll-like receptor 6; TREM2, triggering receptor expressed on myeloid cells 2; TNF-α, tumor necrosis factor alpha. Created in BioRender. Mazarianu, I. (2026) https://BioRender.com/vwdrnx6 (accessed on 25 August 2026). These include Toll-like receptors (TLR2, TLR4 and TLR6), scavenger receptors such as cluster of differentiation 36 (CD36) and scavenger receptor A1 (SR-A1), the receptor for advanced glycation end products (RAGE), TREM2, cluster of differentiation 14 (CD14), cluster of differentiation 47 (CD47), α6β1 integrin and TAM receptor tyrosine kinases [19,24,25]. Recognition of fibrillar Aβ is mediated primarily by the CD36–TLR4–TLR6 receptor complex, which initiates intracellular inflammatory signaling, whereas soluble Aβ oligomers are particularly potent activators of microglial inflammatory responses and neurotoxicity [12]. Following receptor engagement, microglia internalize Aβ through phagocytosis and endocytosis and degrade it via lysosomal pathways [72]. In addition, they secrete proteolytic enzymes, including insulin-degrading enzyme (IDE), neprilysin (NEP), matrix metalloproteinase-9 (MMP-9) and plasminogen, which contribute to extracellular Aβ degradation [21,73]. During the early stages of AD, these mechanisms are largely protective and help limit plaque formation. However, aging and disease progression impair microglial clearance capacity through reduced expression of Aβ-binding receptors CD36, SR-A and RAGE, decreased TREM2 signaling, defective autophagy and increased cellular senescence-associated β-galactosidase activity [19].

The interaction between Aβ, tau and microglia represents a central mechanism driving neuroinflammation in AD (Figure 2). Microglia, the resident immune cells of the CNS, continuously monitor the brain environment and respond to Aβ and tau accumulation through a broad repertoire of pattern-recognition receptors [13].

The APOE isoforms further influence this process, as APOE4 impairs microglial migration toward Aβ deposits and reduces phagocytic uptake compared with APOE3 [74,75]. Beyond Aβ and tau clearance, receptor-mediated Aβ recognition activates intracellular signaling pathways that promote neuroinflammation. Engagement of the CD36–TLR4–TLR6 complex induces nuclear factor kappa-light-chain-enhancer of activated B cells (NF-κB) activation, leading to the transcription of pro-inflammatory genes and priming of the NOD-, IRR- and pyrin domain-containing protein 3 (NLRP3) inflammasome [12,24]. Subsequent signals generated by lysosomal damage, mitochondrial dysfunction, ROS, or potassium efflux trigger assembly of the NLRP3–ASC–caspase-1 complex [76,77]. Activated caspase-1 then promotes the maturation and release of IL-1β and interleukin-18 (IL-18), amplifying inflammatory responses [12]. NLRP3 or caspase-1 deletion in APP/PS1 mice shifts microglia from a pro-inflammatory neurotoxic activation state toward a protective profile, reduces amyloid burden, and improves cognition, confirming that NLRP3 activation is a critical pathogenic node in AD [78]. A consequence of NLRP3 activation is the extracellular release of caspase activation and recruitment domain specks, which can directly bind soluble Aβ and promote its aggregation into amyloid plaques. This establishes a pathogenic feed-forward loop whereby Aβ activates the inflammasome, resulting in ASC release that further accelerates amyloid deposition [79]. Collectively, microglia undergo a progressive transition from a protective to a detrimental phenotype during AD. While early activation supports Aβ clearance and plaque containment through PRR-mediated phagocytosis and the secretion of degrading enzymes, persistent exposure to amyloid drives chronic NF-κB and NLRP3 signaling, excessive cytokine production, oxidative stress and impaired phagocytic function [80]. This self-sustaining inflammatory environment further reduces Aβ clearance efficiency and promotes neuronal dysfunction, synaptic loss and neurodegeneration [27]. Mechanistic investigations in transgenic rodent models have positioned microglia as active mediators of tau propagation rather than passive responders to tau pathology. A landmark preclinical study by Asai et al. demonstrated that pharmacological depletion of microglia in P301S tauopathy mice reduces the spread of pathological tau from the entorhinal cortex to anatomically connected regions, establishing a role for microglia in the trans-neuronal dissemination of tau [81]. Mechanistically, in rodent primary cultures and murine models, microglia internalize extracellular tau aggregates through phagocytosis and subsequently repackage them into exosomes and other extracellular vesicles, which can be taken up by neighboring neurons, thereby facilitating the spread of tau pathology independently of direct synaptic transmission [19,82]. The importance of this pathway is supported by studies showing that inhibition of exosome biogenesis, either through blockade of neutral sphingomyelinase-2 or inhibition of the P2X7 receptor, significantly reduces microglial tau secretion, decreases brain tau accumulation, and improves cognitive performance in the P301S tauopathy mouse model [83]. Furthermore, disease-associated microglia, particularly the microglial neurodegenerative phenotype, appear to play a prominent role in tau dissemination by releasing elevated levels of phosphorylated tau-containing extracellular vesicles [27,82]. These findings suggest that activated microglia not only contribute to neuroinflammation but also act as key facilitators of tau seeding and propagation, providing a mechanistic link between amyloid-driven microglial activation and the progression of tau pathology in AD [27].

### 3.2. Microglia-Astrocyte Interaction in AD

Microglia and astrocytes interact in AD through a bidirectional, self-amplifying signaling network mediated by cytokines, chemokines, complement proteins and growth factors, playing central roles in amyloid plaque phagocytosis, pathological tau clearance and neuroinflammation (Figure 2) [22,84]. Persistent Aβ and tau accumulation drives chronic activation of both cell types, progressively shifting their initially protective responses toward neurodegenerative outcomes [22]. Astrocyte reactivity in AD appears to depend on prior microglial activation, as astrocytic responses to Aβ are markedly reduced in the absence of microglial priming [85]. Aβ activates NF-κB signaling in astrocytes, leading to increased release of complement component C3, which subsequently signals through the C3a receptor (C3aR) expressed on microglia and neurons, thereby modulating neuronal function and microglial activation [24,86]. C3 binding to the neuronal receptor C3aR concurrently disrupts dendritic morphology [87,88]. In parallel, activated microglia release IL-1α, TNF-α and complement C1q (C1q), which drive astrocyte polarization toward the neurotoxic A1 phenotype while reducing their homeostatic and phagocytic functions [86,89]. This microglia-driven astrocyte activation also stimulates β-secretase and γ-secretase activity, cleaving amyloid precursor protein (APP) and stimulating astrocytic Aβ production, thereby augmenting neuronal Aβ and increasing amyloid burden [90]. This cellular crosstalk may exert either protective or detrimental effects depending on the disease stage and pathological context [91]. Acute activation of the C3/C3aR axis can enhance microglial Aβ phagocytosis, whereas chronic activation impairs Aβ clearance and sustains neuroinflammation [89]. Astrocytes also secrete interleukin-3 (IL-3) and, in the presence of Aβ, TREM2-dependent upregulation of interleukin 3 receptor alpha (IL-3Rα) on microglia increases their responsiveness to this signal, promoting microglial motility, plaque clustering, and Aβ and tau clearance [12,86]. However, the effects of glial crosstalk are highly context- and stage-dependent. As AD progresses, prolonged microglial activation promotes persistent cytokine release and activation of kinases such as glycogen synthase kinase-3 beta (GSK-3β), contributing to tau hyperphosphorylation and neurofibrillary tangle formation [92]. Concurrently, astrocytes progressively lose homeostatic functions, leading to a state of “glial paralysis” characterized by impaired neuroprotection, reduced clearance capacity and enhanced neuronal vulnerability, ultimately contributing to neurodegeneration and cognitive decline [93,94,95]. Despite the existence of detrimental inflammatory feedback loops, astrocytes and microglia also act cooperatively to promote Aβ and tau clearance [95,96]. Experimental co-culture studies have shown that interactions between astrocytes and microglia reduce intracellular Aβ accumulation compared with astrocytes alone [97]. Moreover, reactive astrocytes surrounding amyloid plaques contribute to Aβ degradation through increased secretion of proteolytic enzymes such as NEP, endothelin-converting enzyme, IDE, matrix metalloproteinase-2 (MMP2) and MMP9 [98].

## 4. From Phenotypes to Therapeutic Principles

**Figure 3:**
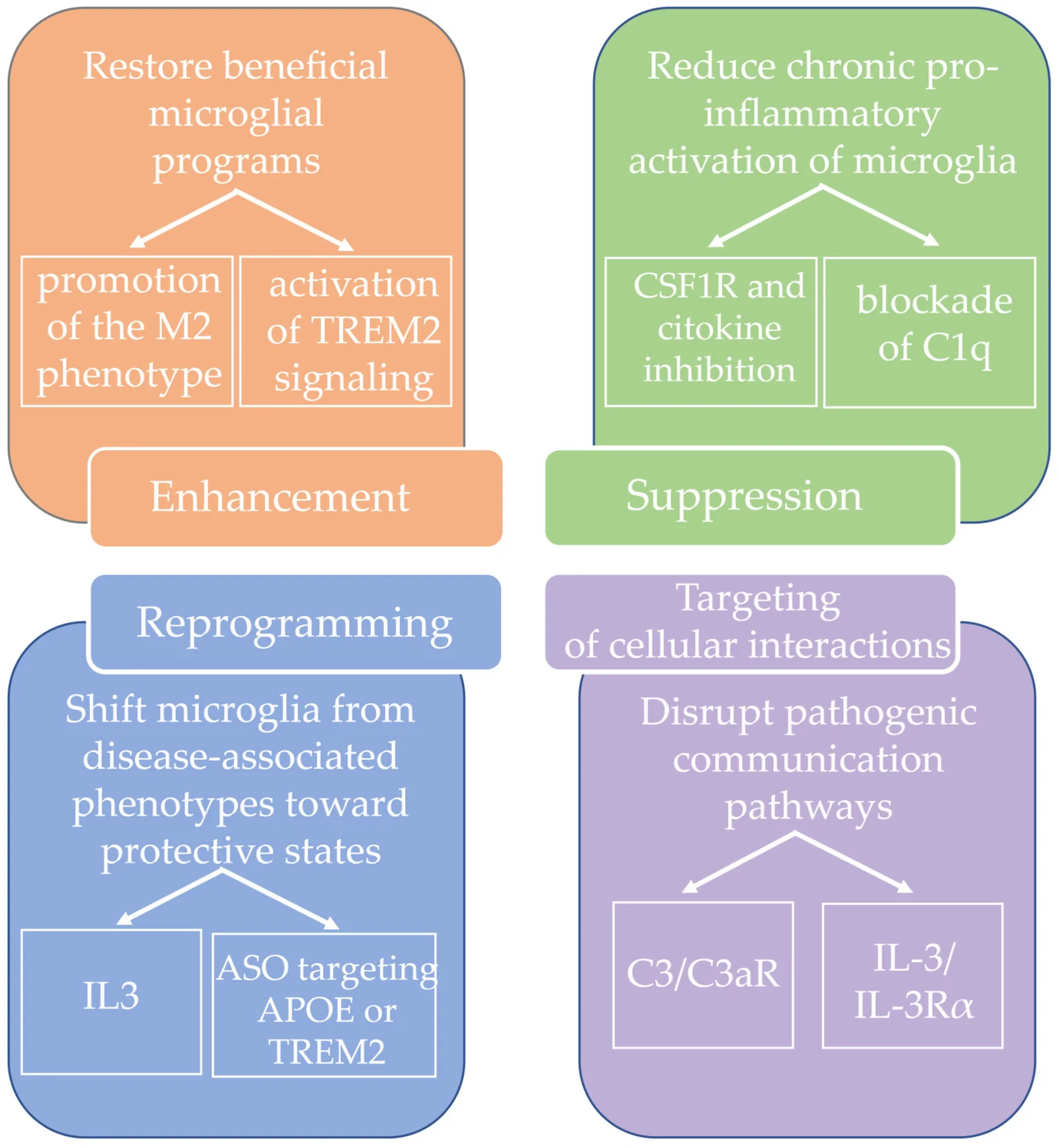
Glial phenotypes can be translated into therapeutic strategies through four principles: enhancement, suppression, reprogramming, and targeting cellular interactions. Abbreviations: interleukin-3 (IL-3), colony-stimulating factor 1 receptor (CSF1R), complement C1q (C1q), triggering receptor expressed on myeloid cells 2 (TREM2), antisense oligonucleotides (ASO), apolipoprotein E (APOE), C3a receptor (C3aR), interleukin-3 receptor alpha (IL-3Rα). Created in BioRender. Mazarianu, I. (2026) https://BioRender.com/koua6xx (accessed on 25 August 2026).

AD-associated microglial signatures should not be uniformly interpreted as detrimental or considered indiscriminate targets for pharmacological inhibition. Instead, emerging evidence supports a context-dependent spectrum of microglial responses that may exert protective and compensatory functions or become maladaptive depending on disease stage and the surrounding pathological environment. During early pathology, reactive populations, including DAM and MGnD states, activate TREM2–APOE-associated programs and accumulate around fibrillar amyloid-β deposits. By forming a cellular barrier around plaques, these microglia can promote plaque compaction, restrict the spread of potentially toxic Aβ species, and reduce damage to neighbouring neurites [43,99]. Thus, maintaining or enhancing these beneficial responses may be preferable to broadly suppressing or eliminating microglia during the initial stages of disease. Microglia may also adopt compensatory programs in response to proteotoxic stress, characterized by increased activity of pathways involved in lipid processing, tissue repair, debris clearance, and inflammatory resolution [56,100]. These adaptations contribute to the preservation of tissue homeostasis and neuronal function, highlighting the potential consequences of indiscriminate microglial depletion or generalized immunosuppression [56]. Conversely, prolonged exposure to amyloid deposits, neuronal damage, and unresolved inflammatory signals can shift microglial responses toward increasingly dysfunctional states [12]. Chronic activation of pathways such as the NLRP3 inflammasome [78], ASC-mediated amyloid seeding [79], and type I interferon signaling may promote complement-dependent synaptic elimination, sustained release of inflammatory mediators, and microglia-associated propagation of tau pathology [81]. At advanced disease stages, persistent cellular stress may further contribute to dystrophic or senescence-like microglial phenotypes, characterized by morphological deterioration and a progressive decline in their homeostatic and neuroprotective functions [101]. Building upon these nuanced functional states, the transition from glial phenotypes to therapeutic strategies can be conceptualized through four major principles: enhancement, suppression, reprogramming and targeting of cellular interactions (Figure 3) [102]. The overall goal is not the complete inhibition of glial activity, but rather the restoration of a context-dependent functional balance [34,103]. Enhancement strategies aim to strengthen beneficial glial programs, including phagocytosis, trophic support and Aβ or tau clearance. Relevant examples include promotion of the protective phenotype [104] and activation of TREM2 signaling [105] all of which have been associated with improved clearance of pathological aggregates and reinforcement of protective glial functions. Suppression approaches focus on reducing chronic detrimental activation, particularly persistent pro-inflammatory states of microglia. Examples include colony-stimulating factor 1 receptor (CSF1R) inhibition [32], blockade of C1q or complement cascade signaling [106], cytokine inhibition [107] and activation of glucagon-like peptide-1 (GLP-1) receptors [108], which collectively reduce inflammatory propagation, synaptic loss and cognitive impairment. Reprogramming strategies aim to shift microglial cells from disease-associated phenotypes toward protective states without eliminating them. For instance, IL-3 signaling can reprogram microglia to enhance clustering around amyloid plaques and promote plaque clearance [86], whereas antisense oligonucleotides (ASOs) targeting APOE or TREM2 can reshape DAM and antigen-presentation signatures [13,96]. Finally, targeting glial interactions focuses on disrupting pathogenic communication pathways, including the C3/C3aR [106], IL-3/IL-3Rα, Plexin-B1 [77], NF-κB [102] and broader complement and cytokine-mediated signaling axes that drive neuroinflammation and neurodegeneration [102]. The central concept is that glial and neuroimmune interactions may initially exert adaptive and neuroprotective effects during the early stages of disease but progressively transition toward maladaptive and neurotoxic responses under conditions of sustained chronic activation [102,109,110]. Consequently, the most effective therapeutic strategies are likely to require precise temporal and pathway-specific modulation of glial signaling networks, rather than broad nonspecific anti-inflammatory interventions [34,86,102].

Microglia-directed interventions should be considered within the broader therapeutic landscape of AD rather than as stand-alone strategies. Phase III trials of the anti-Aβ monoclonal antibodies lecanemab and donanemab in early symptomatic AD demonstrated substantial reductions in brain amyloid burden accompanied by statistically significant slowing of cognitive and functional decline compared with placebo [111,112]. However, the magnitude of clinical benefit remains modest relative to the extent of amyloid removal, and treatment is associated with clinically relevant safety concerns, particularly amyloid-related imaging abnormalities (ARIA) [111,112]. Microglial modulation may provide a complementary therapeutic strategy because microglia participate in antibody-mediated Aβ uptake and plaque clearance. Preclinical studies indicate that disruption of microglial pathways can modify the efficacy of amyloid immunotherapy; in particular, TREM2 deficiency reduces the uptake of antibody-bound Aβ and impairs immunotherapeutic plaque clearance [113]. Consistent with this mechanistic rationale, donanemab has been described as facilitating plaque removal through microglia-mediated phagocytosis, although this mechanism was derived from prior experimental evidence and was not directly evaluated in the phase 3 trial [112]. Importantly, these findings should not be interpreted as evidence that microglia-targeted and anti-amyloid therapies are clinically synergistic. Microglial functions are context-dependent, and experimental depletion studies illustrate potential trade-offs: reducing microglia can attenuate tau propagation while simultaneously increasing amyloid plaque burden and plaque-associated tau pathology [55]. Thus, combination strategies will require careful evaluation of whether microglial modulation enhances beneficial clearance mechanisms without disrupting protective microglial functions or introducing additional toxicity.

A similar rationale applies to emerging tau-directed therapies. Preclinical studies indicate that microglia can influence tau phosphorylation, uptake, release and propagation through inflammatory signaling and extracellular-vesicle pathways [55,114,115]. These findings suggest that selective modulation of microglial pathways could potentially complement therapies that directly target pathological tau. However, this possibility remains mechanistically and preclinically supported rather than clinically established, and the efficacy and safety of combined microglia- and tau-directed interventions require prospective evaluation.

Symptomatic treatments, including cholinesterase inhibitors and memantine, act at a different therapeutic level by providing cognitive and functional benefit without directly targeting the underlying amyloid, tau or microglial pathology [116]. Notably, substantial proportions of participants in the pivotal lecanemab and donanemab trials received concomitant symptomatic AD medications, indicating that disease-modifying anti-amyloid therapy can be administered on a background of established symptomatic treatment; however, these trials were not designed to demonstrate additive or synergistic effects of these combinations [111,112]. More broadly, the Alzheimer’s Tau Platform illustrates the growing clinical interest in multimodal disease-modifying strategies by evaluating tau-directed therapies alone and in combination with anti-amyloid treatment [117]. This approach provides a framework for investigating combination therapy but does not establish the efficacy of adding microglia-directed interventions. Microglia-targeted therapies should therefore be viewed as potential components of stage-specific multimodal treatment strategies whose clinical value will depend on target engagement, preservation of physiological microglial functions, safety, and demonstration of additive clinical benefit in appropriately designed trials.

## 5. Microglia Targeted Therapeutic Strategies and Symptom Modulation

Microglia have emerged as promising therapeutic targets in AD due to their central role in neuroinflammation, Aβ clearance and disease progression. Current therapeutic strategies have evolved from broad suppression of inflammation toward selective modulation of microglial signaling, phenotype and function. These approaches include anti-inflammatory agents, pathway-specific modulation, microglial depletion and replacement, cell- and gene-based therapies, targeted delivery systems and cannabinoid-based interventions. The evidence supporting these therapeutic approaches spans different levels of translational maturity, from mechanistic in vitro studies and preclinical animal models to human observational studies and, for a limited number of strategies, clinical trials. Accordingly, findings from experimental models are interpreted as preclinical evidence and should not be considered indicative of clinical efficacy unless supported by human interventional data. The main strategies, mechanisms and stages of development are summarized in Table 1.

### 5.1. Broad Anti-Inflammatory and Immune-Modulatory Drugs

Nonsteroidal anti-inflammatory drugs (NSAIDs) were among the earliest broad-spectrum anti-inflammatory therapies investigated for AD, based on the hypothesis that chronic neuroinflammation driven by persistent microglial activation and COX signaling contributes to β-amyloid accumulation, tau hyperphosphorylation and synaptic dysfunction [134,162]. Epidemiological studies have consistently identified long-term low-dose aspirin use as the most promising NSAID-related protective factor, showing associations with reduced AD incidence and slower cognitive decline across multiple cohorts, with relative risk reductions ranging from approximately 25% to over 70% [163]. In the review by Peng and Chang (2026), daily low-dose aspirin administration (75–100 mg/day) was associated with a significantly lower dementia risk, with greater protective effects observed following prolonged treatment duration [163]. In contrast, findings for non-aspirin NSAIDs, including ibuprofen, naproxen and selective COX-2 inhibitors, remain inconsistent, ranging from beneficial or neutral effects to evidence of increased neuropathological burden at high cumulative doses. For example, Sonnen et al. (2010) reported that cumulative NSAID exposure exceeding 1000–2000 standard daily doses was associated with increased neuritic plaque pathology [164], while selective COX-2 inhibitors generally failed to demonstrate efficacy in either experimental or clinical studies [134]. Collectively, current evidence suggests a possible protective effect of long-term low-dose aspirin in selected elderly populations, although no uniform neuroprotective class effect can be established for all NSAIDs. Importantly, discrepancies between epidemiological observations and randomized clinical trial outcomes may largely reflect methodological limitations. Several trials, including the Alzheimer’s Disease Anti-inflammatory Prevention Trial (ADAPT) [165], enrolled older participants with already established neuropathology (mean age ≥ 70 years), applied relatively short treatment durations (<2 years), and frequently used subtherapeutic dosing regimens [163]. NSAIDs exert multiple effects on microglia and astrocytes in AD beyond classical COX inhibition. Their canonical action involves inhibition of COX-1 and COX-2, reducing prostaglandin-mediated neuroinflammation [134]. COX-1 is predominantly expressed in activated microglia and appears to be a major driver of inflammatory signaling in AD, whereas neuronal COX-2 is more closely associated with synaptic dysfunction and excitotoxicity [166]. Accordingly, non-selective NSAIDs such as ibuprofen and indomethacin inhibit both isoforms, while selective COX-2 inhibitors have generally failed in AD clinical trials. Experimental studies further demonstrate that ibuprofen, indomethacin and flurbiprofen derivatives reduce Aβ plaque burden, suppress microglial and astrocytic activation, and decrease pro-inflammatory cytokines including IL-1β and TNF-α [135,166]. In parallel, NSAIDs such as ibuprofen and indomethacin activate PPARγ signaling, suppressing microglial release of cytokines, inducible nitric oxide synthase, and other inflammatory mediators while also enhancing Aβ phagocytosis and repressing beta-site amyloid precursor protein cleaving enzyme 1 (BACE1) expression [166]. Aspirin and sodium salicylate additionally inhibit NF-κB signaling, thereby reducing glial inflammatory activation and NLRP3 inflammasome-related pathways [167]. NSAIDs also attenuate astrocyte reactivity and partially restore hippocampal neurogenesis in experimental rat models [168], although these anti-inflammatory effects appear most effective when initiated during early disease stages before chronic glial activation becomes established, as Lim et al. (2000) demonstrated in the Tg2567 mice AD model [169].

Translational status and limitations: collectively, current evidence suggests a possible protective effect of long-term low-dose aspirin in selected elderly populations, although no uniform neuroprotective class effect can be established for all NSAIDs. Importantly, discrepancies between epidemiological observations and randomized clinical trial outcomes may largely reflect methodological limitations. Several trials, including the ASPREE Trial (NCT01038583), enrolled cognitively intact older participants (mean age ≥ 70 years) who received daily administration of 100 mg enteric-coated aspirin for a median follow-up of 4.7 years. Aspirin failed to reduce the incidence of all-cause dementia, mild cognitive impairment, or persistent cognitive decline, while significantly increasing the risk of major gastrointestinal and intracranial hemorrhages [170]. The ADAPT (NCT00007189), which evaluated naproxen and celecoxib in older participants (mean age ≥ 70 years), was terminated early because of cardiovascular safety signals and showed no cognitive protection over extended follow-up [165]. However, an important limitation remains CNS penetration, as many NSAIDs (e.g., flurbiprofen and enantiomers of ibuprofen) achieve only low cerebrospinal fluid concentrations. Escalating dosages to overcome limited CNS exposure is severely restricted by peripheral toxicities [166]. Sustained systemic inhibition of cyclooxygenases in elderly, frail populations markedly elevates the incidence of life-threatening gastrointestinal ulcerations, renal dysfunction, and adverse cardiovascular events [171]. Preclinical animal models consistently demonstrate therapeutic benefit only when anti-inflammatory agents are administered during early, pre-symptomatic windows before chronic glial activation consolidates [169]. In contrast, clinical cohorts enroll patients with already established neuropathological cascades, where secondary senescent or exhausted microglial states no longer respond to broad cyclooxygenase inhibition [165]. Consequently, heterogeneous clinical outcomes likely reflect differences in glial target engagement, CNS bioavailability, pathway specificity, and timing of intervention relative to disease progression. Early intervention with anti-inflammatory agents in presymptomatic or mild cognitive impairment cohorts with low baseline amyloid burden may successfully restrain initial microglial hyperactivation and subsequent plaque seeding, whereas applying the same treatment in advanced, dementia-stage patients bearing established dystrophic microglia yields minimal cognitive benefit while exposing frail populations to systemic bleeding and cardiovascular risks [42].

### 5.2. Minocycline, PPAR Agonists and Other Small Molecules

Minocycline, a second-generation semi-synthetic tetracycline antibiotic [138], has attracted considerable interest as a potential therapeutic agent in AD because of its anti-inflammatory, antioxidant, anti-apoptotic, and neuroprotective properties [136,172]. It efficiently penetrates the blood–brain barrier (BBB) and CSF, allowing direct modulation of neuroinflammatory pathways within the CNS [173]. In primary rodent glial cultures and immortalized BV-2 cell assays, minocycline suppresses microglial reactivity, inhibits matrix metalloproteinases (MMPs) [136], and reduces pro-inflammatory mediators [174], including IL-1β, IL-6, and TNF-α [175]. When evaluated in transgenic APP murine models, minocycline administration attenuated cholinergic neuronal degeneration and improved behavioral performance in spatial memory tasks, although it failed to alter insoluble Aβ plaque burden [138].

Peroxisome proliferator-activated receptors (PPARs) are ligand-activated transcription factors of the nuclear hormone receptor superfamily, comprising three isoforms: PPARα, PPARβ/δ, and PPARγ [176,177]. These receptors regulate lipid and glucose metabolism, cellular differentiation, proliferation, and inflammatory responses [177]. In the CNS, PPARs are expressed in microglia and astrocytes, but under AD physiological conditions, PPARγ mRNA expression in the adult brain is relatively low and mainly restricted to hippocampal dentate gyrus granule cells, with lower expression in the caudate putamen, globus pallidus, thalamus, and piriform cortex, supporting its involvement in neuroinflammatory and neurodegenerative processes [140]. PPARγ activation as a regulator of CNS metabolic and inflammatory functions in both physiological and pathological conditions emerged largely from studies investigating NSAIDs in AD. NSAIDs were shown to reduce neuronal vulnerability through inhibition of COX-2, a major mediator of inflammatory cascades associated with AD pathology [134]. Subsequently, activation of PPARγ in macrophages and glial cells was found to suppress the expression of pro-inflammatory mediators such as TNF-α and iNOS, thereby limiting inflammatory damage and contributing to cognitive improvement in AD [178]. PPARγ signaling also plays a central role in suppressing pro-inflammatory polarization and activating neuroprotective transcriptional pathways [179]. Preclinical studies in primary cell cultures further demonstrated that the PPARγ activator SNU-BP suppressed iNOS activity and pro-inflammatory cytokine production in LPS-stimulated microglia while increasing interleukin-4 (IL-4) and ARG1 expression, consistent with neuroprotective transcriptional microglial pathways [180]. Similarly, pioglitazone-treated APP/PS1 mouse model showed reduced microglial activation surrounding amyloid plaques, increased protective marker expression, decreased glial fibrillary acidic protein (GFAP)-positive astrocytes, and enhanced astrocytic internalization of Aβ [178]. The small-molecule agonist DSP-8658 has been shown to enhance microglial phagocytosis while promoting polarization toward the anti-inflammatory phenotype, in animal AD models [181]. Kummer et al. demonstrated that the pan-PPAR agonist GFT1803, which activates all PPAR isoforms, significantly reduced Aβ plaque burden, Aβ levels, and microglial activation in APP/PS1 mouse models. These effects were associated with enhanced Aβ degradation and clearance, partly through increased expression and activation of IDE [182]. In experimental models, the PPARα agonist gemfibrozil significantly decreased amyloid plaque deposition and microgliosis in 5XFAD mice, while also improving spatial memory and learning; notably, these beneficial effects were absent in PPARα-deficient mice [183]. PPARβ/δ agonist GW0742, in a mouse AD model, attenuated Aβ-induced hippocampal neurotoxicity by restoring PPARβ/δ expression, reducing NF-κB p65 activation, TNF-α, IL-1β, and cleaved caspase-3 levels, while simultaneously increasing the anti-apoptotic B-cell lymphoma 2 (Bcl-2)/BCL2 associated X (Bax) ratio [184]. In addition to its anti-inflammatory properties, PPARβ/δ contributes to cerebrovascular homeostasis by enhancing vascular endothelial growth factor (VEGF) receptor expression and endothelial nitric oxide production [185].

Additional small molecules are currently being investigated for their capacity to modulate neuroinflammation through more selective and pathway-specific mechanisms, though current evidence remains predominantly restricted to in vitro platforms and early preclinical models. Pterostilbene attenuates neuroinflammation by inhibiting the NLRP3/caspase-1 inflammasome pathway and reducing IL-1β secretion in the BV-2 microglial cell line [25,186], while edaravone, a BBB-permeable free radical scavenger, suppresses Aβ-induced microglial activation in primary rodent cultures through inhibition of NLRP3 inflammasome-mediated IL-1β release [187,188]. Benzyl isothiocyanate, a compound found in cruciferous vegetables [25], similarly inhibits inflammasome activation and pro-inflammatory cytokine production in microglia [25,27]. Curcumin has also demonstrated anti-inflammatory and neuroprotective effects in AD animal models through regulation of glial activation and reduction in AD-related pathology [185]. In parallel, several kinase inhibitors, including p38 mitogen-activated protein kinase (MAPK) inhibitors, nilotinib, baricitinib, and fasudil [82], are being explored in preclinical animal setups for their capacity to suppress pro-inflammatory microglial phenotypes [24], regulate neuroinflammatory signaling, and modulate microglial metabolism [82]. Whether these cellular and murine findings can produce meaningful disease-modifying effects in human clinical neurodegeneration without causing intolerable systemic immunosuppression remains to be determined.

Translational status and limitations: despite promising results in experimental models, the translation of minocycline, PPAR agonists, and other small-molecule immunomodulators to human clinical practice has faced notable roadblocks. In human clinical trials, the Phase II MADE (NCT01463384) with minocycline in mild-to-moderate AD cohorts failed to replicate these cognitive benefits and showed no meaningful slowing of disease progression [137,139]. Similarly, although agents such as pioglitazone and rosiglitazone improved amyloid clearance and cognitive performance in transgenic animal studies, they failed to demonstrate significant efficacy in Phase III human clinical trials [24,95]. The REFLECT program (NCT00428090) evaluating rosiglitazone in mild-to-moderate AD cohorts showed no improvement in primary cognitive endpoints [189,190], and the large-scale TOMMORROW prevention trial (NCT01931566) with low-dose pioglitazone in cognitively unimpaired individuals with high genetic risk (APOE and TOMM40 genotypes) was terminated early due to lack of efficacy [127,191]. This lack of clinical benefit has been largely attributed to poor blood–brain barrier permeability, resulting in insufficient CNS bioavailability [178]. Moreover, the high doses required to achieve therapeutic brain concentrations raised important safety concerns, including weight gain, hepatic abnormalities, edema, and increased risk of heart failure [178,192]. For novel candidate molecules and kinase inhibitors, achieving therapeutic central concentrations that suppress microglial activation without causing intolerable systemic immunosuppression remains to be clinically resolved. More fundamentally, these translational failures highlight the limitation that rodent models capture acute, amyloid-driven microglial phenotypes that respond readily to metabolic reprogramming, whereas patients in clinical trials already exhibit chronic neurodegeneration, extensive synaptic loss, and senescent microglial changes that may no longer be responsive to isolated PPARγ modulation [136]. Metabolic reprogramming through PPARγ agonism or minocycline appears therapeutically viable only during the prodromal phase in non-demented patients, where preserving baseline microglial phagocytosis and halting nascent neuroinflammation are still achievable, whereas the same interventions fail to rescue advanced dementia patients whose chronically primed and senescent microglia no longer respond to metabolic stimulation.

### 5.3. NLRP3 Inflammasome and Cytokine Targeting

Microglial inflammasome activation, particularly through the NLRP3 pathway, is increasingly recognized as a central driver of AD pathology and a major mediator of chronic neuroinflammation [76]. The NLRP3 inflammasome is a multiprotein complex composed of NLRP3, the adaptor protein ASC and pro-caspase-1 [187] and its activation occurs predominantly in plaque-associated microglia [77]. NLRP3 activation in microglia occurs through two sequential phases: priming and activation [193]. During the priming phase, aggregated Aβ binds to the scavenger receptor CD36 and forms complexes with TLR4 and TLR6 on the microglial surface, triggering NF-κB signaling and promoting transcription of NLRP3 and IL-1β [194]. In the activation phase, fibrillar Aβ uptake induces lysosomal rupture, cathepsin B release, mitochondrial dysfunction, and ROS generation, all of which promote inflammasome assembly and pro-caspase-1 activation [195]. Activated pro-caspase-1 subsequently drives the maturation and secretion of IL-1β and IL-18, amplifying neuroinflammatory responses and neuronal injury [77]. NLRP3 activation shifts microglia toward a pro-inflammatory phenotype characterized by elevated caspase-1 and IL-1β expression, increased amyloid deposition, neuronal damage and cognitive impairment [78]. Conversely, deletion of NLRP3, ASC, or caspase-1 in APP/PS1 transgenic mice promotes a more protective and phagocytic microglial phenotype, enhances Aβ clearance, reduces amyloid burden, attenuates tau pathology, and improves cognitive performance [12]. In preclinical transgenic rodent models (APP/PS1, 5XFAD and J20 mice), pharmacological inhibitors such as MCC950 [144], JC-124 [78], CY-09, VX-765 [76], and related compounds consistently suppress inflammasome activation, reduce Aβ accumulation, enhance microglial phagocytosis, attenuate tau hyperphosphorylation or aggregation, and improve cognitive outcomes in behavioral assays [76,143,144]. Mechanistically, MCC950 and CY-09 inhibit NLRP3 activation by targeting the NACHT domain and preventing ATP hydrolysis required for inflammasome oligomerization [77] whereas VX-765 acts downstream as a selective caspase-1 inhibitor, reducing neuroinflammation, preserving synaptic integrity, and limiting Aβ-associated pathology in AD mouse models [196]. However, evidence supporting NLRP3 inhibition in AD remains predominantly preclinical. Although MCC950 reduced neuroinflammation, Aβ accumulation and cognitive impairment in a murine model of sporadic AD, its therapeutic efficacy and safety in human AD remain to be established [197].

Translational status and limitations—clinical translation of the NLRP3–caspase-1–cytokine pathway in human AD cohorts is marked by a clear dichotomy between preclinical prototypes and clinical-stage downstream candidates. Direct, selective small-molecule NLRP3 inhibitors (such as MCC950, JC-124, and CY-09) remain strictly restricted to preclinical investigations, with prototypical candidates like MCC950 being discontinued prior to clinical development due to severe hepatotoxicity [198]. In contrast, therapeutic modulation of downstream inflammasome nodes has advanced into human Phase II clinical trials: the caspase-1 inhibitor VX-765 underwent Phase II testing in humans with treatment-resistant epilepsy (demonstrating safety and target engagement, though primary efficacy endpoints were not achieved in epilepsy), while the anti-IL-1β monoclonal antibody canakinumab has progressed to Phase II evaluation in patients with mild dementia due to AD (NCT04795466) [199,200,201]. Furthermore, biologics directed against downstream effectors, including the IL-1 receptor antagonist anakinra and the anti-IL-1β monoclonal antibody canakinumab (under evaluation in phase II clinical trials in patients with mild dementia due to AD), face limited central nervous system penetrance across the intact human BBB [201]. Therefore, while preclinical rodent studies provide compelling proof-of-concept that dampening the NLRP3 cascade halts disease progression, clinical verification in human randomized trials is essential to establish whether small-molecule inflammasome blockers can achieve therapeutic central concentrations without inducing intolerable systemic immunosuppression [202]. Blunting the NLRP3–caspase-1 axis exerts stage-divergent benefits: in early asymptomatic stages, it halts ASC speck-mediated amyloid nucleation, whereas in moderate-to-severe disease with confirmed tau spreading, the same treatment acts predominantly by arresting cytokine-driven synaptic loss and tau propagation, rendering biomarker-positive patients with elevated CSF/plasma neuroinflammatory metrics (such as GFAP and IL-1β) the ideal target subpopulation [79].

### 5.4. Pathway-Specific Modulation: TREM2, APOE and Complement

Triggering receptor expressed on myeloid cell 2 (TREM2) is a microglia-enriched innate immune receptor belonging to the immunoglobulin-lectin-like receptor superfamily and plays a central role in regulating microglial activation and function in AD [203] and binds APOE with high affinity, linking the two strongest genetic risk factors for late-onset AD into a unified signaling pathway [204]. TREM2 associates with the transmembrane adaptor DAP12 (TYROBP), and the TREM2–DAP12 complex transduces intracellular signals through immunoreceptor tyrosine-based activation motif (ITAM) phosphorylation and activation of the SYK kinase pathway [205]. Through this signaling cascade, TREM2-APOE pathway regulates microglial processes and the transition toward the DAM/MGnD phenotype during neurodegeneration. TREM2 recognizes multiple ligands, including phospholipids, sulfatides, lipoproteins and APOE, particularly on apoptotic or damaged cells. Through these interactions, TREM2 promotes microglial phagocytosis, survival, and proliferation while helping to limit excessive TLR-mediated inflammatory responses [53]. Loss or deficiency of TREM2 arrests microglial activation at the TREM2-independent early DAM stage, preventing transition into the protective, highly phagocytic TREM2-dependent mature DAM state, resulting in more diffuse Aβ plaques, increased neuronal exposure to toxic amyloid species and enhanced axonal damage [12]. In contrast, TREM2 promotes microglial-mediated plaque compaction, limiting plaque diffusion and reducing amyloid-associated neurotoxicity [99]. Consistently, TREM2 overexpression in 5xFAD mice enhances microglial phagocytosis and reduces Aβ deposition [150]. The effects of TREM2 in AD appear to be highly stage-dependent. Early TREM2 deficiency may transiently reduce amyloid accumulation and limit excessive synaptic phagocytosis, suggesting potential neuroprotective effects during initial disease stages [206]. However, at later stages, loss of TREM2 exacerbates synaptic dysfunction and cognitive decline by impairing microglial phagocytosis of both synapses and Aβ plaques [207]. Although TREM2 is generally considered protective in amyloid pathology, its role in tauopathies remains controversial. Several studies showed that TREM2 deficiency or the R47H variant enhances tau seeding and spreading around amyloid plaques, whereas other reports found that TREM2 loss reduced brain atrophy and microglial activation in tau transgenic models [208,209]. These findings suggest that the impact of TREM2 on both Aβ and tau pathology may vary ac-cording to disease stage and pathological context. Given its regulatory role in microglial responses, TREM2 has emerged as a therapeutic target in AD. VHB937, a TREM2-targeting antibody, has been shown to enhance chemotaxis and phagocytic activity in vitro and in AD animal models, demonstrating neuroprotective effects, including preservation of neuronal viability and reduction in neuroinflammation [122]. Unlike antibody-based TREM2 activators, VG-3927 is an orally available small-molecule TREM2 agonist, which in 5xFAD mice, decreased amyloid plaque burden and insoluble Aβ42 levels while promoting a DAM-like transcriptional profile [120].

APOE is the primary ligand for TREM2 on microglia and together they form a unified signaling axis that links the two strongest genetic risk factors for late-onset AD [74]. The APOE gene exists in three common isoforms: ε2 (protective), ε3 (neutral) and ε4 (risk), with APOE4 conferring a 3- to 4-fold increased risk in heterozygotes and up to 15-fold in homozygotes, while APOE2 reduces risk by nearly half [50,210]. In the healthy brain, APOE is predominantly produced by astrocytes, but its expression is markedly upregulated by reactive microglia surrounding amyloid plaques as part of the conserved DAM/MGnD transcriptional program [211]. This microglial APOE feeds back through TREM2 to sustain the activated phenotype and the TREM2-APOE pathway was identified as the master regulator of the microglial switch from homeostasis to neurodegeneration-associated states [56]. Genetic ablation of either TREM2 or APOE in APP-PS1 mice restored homeostatic microglial signatures and in tauopathy models, arrested neurodegeneration, demonstrating that this axis is required to drive the dysfunctional microglial response [12,32]. Evidence that APOE4 contributes to the course of AD through both loss-of-function and gain-of-toxic-function processes impacting microglial, vascular, and neuronal pathways has led to the emergence of APOE-targeted treatment techniques as a promising strategy. Several major therapy modalities can be used to broadly classify current techniques [74,211]. The increased risk associated with APOE4 is thought to arise, at least in part, from structural differences between APOE isoforms. A single amino acid substitution (Arg112 instead of Cys112) alters the conformation of APOE4, promoting a domain interaction between its N- and C-terminal regions that may contribute to its pathogenic effects in AD [212]. A growing number of therapeutic strategies are being developed to target APOE4-driven pathology in AD. These approaches range from restoring normal APOE function through structure correction and lipidation enhancement to reducing APOE4 expression or modifying its biological activity via immunotherapeutic and genetic interventions. Current APOE-directed approaches range from exploratory preclinical molecules to clinically tested mimetic peptides. At the in vitro and cellular level, the small molecule GIND25 (Azocarmine G) disrupts pathological APOE4 domain interactions to correct it, promotes neurite outgrowth and synaptic development, reduces Aβ accumulation, and restores mitochondrial cytochrome c oxidase subunit 1 (mtCOX1) expression in cultured neuronal cells [213]. Similarly, the small-molecule CBA2 relieved APOE4-dependent repression of autophagy and lysosomal gene networks in cellular models, thereby facilitating intracellular Aβ clearance [214], both remain strictly in preclinical research. In contrast, approaches enhancing APOE lipidation through modulation of the ATP-binding cassette transporter A1 (ABCA1) by the APOE mimetic peptide CS-6253 reduced glial lipid accumulation promoted Aβ and tau clearance and improved both vascular function and cognitive performance in preclinical studies and has advanced to clinical evaluation, where a Phase I trial (NCT05965414) demonstrated favorable safety and pharmacokinetic profile [124]. In preclinical transgenic rodent models, inhibition of diacylglycerol acyltransferases (DGAT1/2) reduces DGAT2-dependent lipid droplet accumulation in microglia, restoring microglial Aβ phagocytosis, decreasing amyloid plaque burden, and attenuating neuronal damage in 5xFAD mice [215]. The anti-APOE antibody HJ6.3 promoted the recruitment of cluster of differentiation 45 (CD45)-positive microglia to amyloid plaques while reducing inflammatory cytokine production in preclinical mouse model APPswe/PS1ΔE9 [216]. Similarly, HAE-4, an antibody selective for the APOE4 isoform, enhanced microglial accumulation around APOE-positive Aβ deposits and improved amyloid clearance in transgenic 5XFAD mice expressing human APOE4 [217]. Antisense oligonucleotides targeting APOE4 mRNA have been shown to preserve synaptic integrity, reduce microglia-mediated synaptic phagocytosis, and lower plasma neurofilament light chain levels, indicating reduced neurodegeneration in murine tauopathy models (P301S mice) [218].

Translational status and limitations—despite compelling genetic validation and preclinical efficacy, clinical translation of TREM2- and APOE-directed therapeutics into human cohorts has encountered substantial obstacles across pharmacokinetic, biological, and clinical domains. The failure of the humanized agonistic TREM2 antibody AL002 highlights the difficulty of translating microglial receptor activation into clinical efficacy. In the first-in-human Phase I INVOKE-1 study, intravenously administered AL002 was measurable in cerebrospinal fluid (CSF), with CSF-to-serum partition coefficients of approximately 0.07–0.28%, and produced dose-dependent reductions in CSF soluble TREM2, providing direct evidence of CNS exposure and pharmacodynamic target engagement [219]. However, these findings did not establish clinical efficacy. In the Phase II INVOKE-2 randomized clinical trial (NCT04592874) involving patients with early AD, AL002 failed to meet its primary cognitive endpoint or secondary biomarker endpoints and did not achieve significant amyloid plaque reduction on PET imaging [76,220]. While oral small-molecule agonists VG-3927 has entered Phase I clinical evaluation to establish its central nervous system penetrance, pharmacokinetics, and safety in humans, clinical proof-of-concept for small-molecule TREM2 activation in patient cohorts remains unproven [121]. Biologic monoclonal antibodies targeting microglial surface receptors face notoriously low blood–brain barrier penetration, with central antibody concentrations typically reaching less than 0.1% of systemic circulating levels [221]. Furthermore, animal studies reveal that the physiological role of TREM2 shifts dynamically across the disease continuum [222]. Broad target engagement in advanced disease stages may fail if microglial responsiveness has already undergone dystrophic exhaustion or if receptor stimulation during established tau propagation inadvertently accelerates tau-mediated neurodegeneration [42]. Clinical efforts to enhance APOE lipidation pharmacologically have also highlighted the divergent responses between human APOE genotypes. In a Phase II proof-of-concept trial in moderate AD patients (NCT01782742), the RXR (retinoid X receptor) agonist bexarotene failed to lower amyloid burden overall, but post hoc analyses revealed modest amyloid clearance exclusively in APOE4 non-carriers, accompanied by drug-induced hypertriglyceridemia. The synthetic APOE-mimetic peptide CS-6253, which stimulates ABCA1-mediated lipidation and aggregate clearance in animal models, completed a Phase I clinical trial (NCT05965414) to establish safety, tolerability, and pharmacokinetics in healthy human volunteers and its cognitive efficacy and microglial impact in AD patients remain to be evaluated in Phase II cohorts [223]. Beyond direct myeloid targeting, exploratory findings suggest that peripheral lipid modulation may indirectly influence circulating neurodegenerative metrics. In a pre-specified secondary biomarker sub study of the Phase III cardiovascular BROADWAY trial (NCT05142722), the cholesteryl ester transfer protein inhibitor obicetrapib was associated with attenuated progression of plasma phosphorylated tau (p-tau217) and GFAP, predominantly in older cardiovascular patients carrying the APOE4 allele [224,225]. Collectively, because this trial evaluated cardiovascular endpoints in non-demented participants, these exploratory circulating biomarker observations must not be overstated as direct microglial modulation or cognitive disease modification. However, dedicated randomized trials in clinically diagnosed, symptomatic AD cohorts remain necessary to determine whether these peripheral lipid-modulating effects translate into cognitive preservation. Agonistic TREM2 activation or APOE-directed therapies provide maximal utility when administered in early mild cognitive impairment, particularly in APOE4 carriers, by reinforcing microglial plaque barrier compaction, whereas identical stimulation in advanced stages with established tau pathology risks exacerbating tau dissemination within exhausted myeloid niches. Clinical trial evaluations across human cohorts demonstrate that microglial-targeted therapies such as TREM2 activation or APOE modulation exert stage-divergent benefits through distinct mechanisms: in early prodromal or mild cognitive impairment stages, these interventions help primarily by stabilizing neurodegenerative biomarker cascades and correcting local lipid metabolism, whereas in moderate-to-advanced clinical dementia, the same interventions serve predominantly to blunt secondary neuroinflammatory surges rather than reverse established amyloid or tau burden [223,224,225].

### 5.5. Microglial Depletion and Replacement

#### 5.5.1. CSF1R Inhibition and Microglial Depletion

CSF1R inhibition has emerged as a strategy to transiently deplete resident microglia and reshape the neuroinflammatory environment in AD. Because CSF1R is essential for microglial survival and proliferation, pharmacological inhibitors such as PLX3397 (pexidartinib) and PLX5622 can deplete approximately 70–90% of resident microglia in the CNS [131]. Studies conducted by Spangenberg et al. in a preclinical mouse model, 5XFAD, also revealed that microglia contribute to the compaction of Aβ plaques; consequently, their ablation can alter plaque morphology from dense core to more diffuse plaques, increasing neuronal exposure to toxic amyloid species [100]. Therapeutic benefits of CSF1R inhibition arise primarily from microglial repopulation rather than prolonged depletion itself [226]. Following withdrawal of CSF1R inhibitors, a small population of surviving microglia rapidly proliferates and repopulates the CNS [227]. These newly generated microglia display morphology, density and transcriptional signatures similar to those of homeostatic microglia, effectively replacing dysfunctional, chronically activated cells [29,148]. Repopulated microglia have been shown to exhibit reduced inflammatory activation, improved phagocytic function and enhanced support of neuronal homeostasis [148]. In several preclinical models of neurodegeneration, traumatic brain injury, and aging, microglial repopulation was associated with reduced neuroinflammation, enhanced neurogenesis, restoration of synaptic plasticity, and improved cognitive function [227]. In aged transgenic amyloid mouse models, repopulated microglia restored microglial morphology and density to more youthful states, normalized age-related gene expression patterns, increased dendritic spine density, and improved hippocampal long-term potentiation [228]. These observations suggest that microglial replacement may serve as a means of “rejuvenating” the microglial population and restoring a more protective phenotype. However, CSF1R-mediated microglial depletion may have opposing effects on AD pathology. In AppNL-G-F mice, PLX5622-mediated microglial depletion reduced tau propagation but simultaneously increased amyloid plaque burden and plaque-associated tau-positive dystrophic neurites [55]. Nevertheless, the effects of microglial depletion remain highly context-dependent. Complete elimination of microglia can be detrimental in certain conditions, reflecting their essential roles in immune surveillance, synaptic maintenance and tissue repair [45,227].

Translational status and limitations—while experimental rodent models demonstrate therapeutic benefits, clinical translation of CSF1R-mediated microglial depletion is severely hindered by systemic toxicity, species-specific renewal kinetics, and uncharacterized biological risks in human patients. Because CSF1R is essential for microglial survival and proliferation, pharmacological inhibitors such as PLX3397 (pexidartinib) can deplete approximately 70–90% of resident microglia in the CNS in preclinical research. Although pexidartinib has undergone clinical evaluation and received regulatory approval in human oncology for tenosynovial giant cell tumors in a Phase III clinical trial, its translational feasibility for chronic neurodegenerative indications is critically constrained by severe systemic toxicities [229]. Pexidartinib carries an FDA boxed warning for potentially fatal, mixed or cholestatic drug-induced liver injury, alongside marked neutropenia and peripheral myelosuppression resulting from the obligatory dependence of bone marrow-derived myeloid precursors on intact CSF1R signaling [230]. Translating systemic CSF1R inhibition into human AD therapeutics faces substantial biological and pharmacological roadblocks. The kinetics of microglial repopulation differ markedly between species: while the rodent central nervous system rapidly refills the microglial niche within 7 to 14 days following inhibitor cessation [231], human microglial renewal is exceptionally slow, characterized by an estimated median turnover time of several decades [16]. Consequently, sustained ablation in elderly patients risks precipitating prolonged, severe myeloid void within the brain parenchyma, predisposing the central nervous system to opportunistic infections and unconstrained amyloid seeding [145].

Rather than producing a uniform biological effect, pharmacological CSF1R inhibition operates through divergent mechanisms across disease phases: in nascent or prodromal stages, myeloid depletion proves detrimental by dismantling the physical microglial barrier required to compact fibrillar cores, thereby heightening parenchymal exposure to diffuse, neurotoxic amyloid species [100]. Conversely, in late-stage pathology driven by chronic microglial reactivity and secondary synapse loss, identical CSF1R inhibition acts via pulse-depletion regimens that purge exhausted, pro-inflammatory populations to permit the engraftment or repopulation of homeostatic cells [16,148]. Accordingly, clinical-stage candidate stratification should exclude early amyloid-positive, non-demented individuals and instead prioritize symptomatic cohorts exhibiting established neuroinflammatory signatures, who require targeted clearance of a chronically primed, senescent microglial pool.

#### 5.5.2. Microglial Replacement Strategies and Senescence Targeting

Brain aging is a major risk factor for AD and other neurodegenerative disorders, and age-related microglial dysfunction is increasingly recognized as a main contributor to disease progression [232,233]. Aged microglia exhibit impaired phagocytosis, reduced motility, altered immune signaling, defective autophagy and diminished clearance of Aβ, myelin debris and apoptotic cells [234]. These changes promote the accumulation of toxic protein and lipid aggregates, further compromising neuronal and microglial health. Additionally, senescent microglia display dystrophic morphology, increased expression of senescence markers (p16, p21, p53, SA-beta-gal) and a pro-inflammatory phenotype [235]. Preclinical rodent models have shown that factors derived from young microglia can restore Aβ clearance in aged microglia, while systemic rejuvenation approaches such as parabiosis or administration of young plasma improve neurogenesis, synaptic plasticity, and cognition in aged animals [236]. Similarly, myeloid growth factor modulation has advanced as a pro-clearance paradigm: granulocyte-macrophage colony-stimulating factor (GM-CSF) stimulates microglial proliferation, promotes phagocytic aggregate clearance, and reverses hippocampal dendritic loss in amyloid murine models [237]. Microglial replacement has emerged as another potential rejuvenation approach in preclinical rodent studies. Following depletion and repopulation, newly generated microglia in aged mice restore microglial density and morphology, normalize aging-related gene expression (reversing 127 age-dysregulated genes including *Apoe*, *Bmp6*, and *Sorl1*), improve synaptic function, and enhance cognitive performance [238]. Although the aged brain environment can still impose some pro-inflammatory features on repopulated cells, overall evidence indicates that aged microglia retain significant rejuvenation potential, highlighting microglial repair and replacement as promising therapeutic avenues for neurodegenerative diseases [45,238].

Translational status and limitations—translating microglial rejuvenation, growth factor modulation, and replacement paradigms into human clinical applications involves distinct hurdles across clinical trial design, delivery logistics, and biological sustainability. Among myeloid growth factor strategies, GM-CSF has transitioned into clinical evaluation; in a randomized, double-blind Phase II trial (NCT01409915), treatment with recombinant human GM-CSF (sargramostim) demonstrated acceptable safety and yielded preliminary, statistically significant improvements in Mini-Mental State Examination scores alongside modulation of plasma neurodegenerative biomarkers in mild-to-moderate AD patients [99]. Broad implementation of microglial replacement and senolytic strategies faces critical pharmacological and host-environment constraints. Cell-replacement approaches introduce cells into an intrinsically hostile brain milieu characterised by dense amyloid plaques, tau neurofibrillary tangles, chronic oxidative stress, and reactive astrogliosis. Without concurrent remodeling of this established microenvironment, newly engrafted or repopulated microglia risk rapid phenotypic exhaustion, reverting to dystrophic and dysfunctional states [42]. In asymptomatic or early mild cognitive impairment stages, microglial rejuvenation or growth-factor stimulation (e.g., GM-CSF) helps primarily by reinforcing baseline surveillance, preventing early autophagic failure, and sustaining non-inflammatory clearance of nascent aggregates before cellular priming occurs [233,239], whereas in moderate-to-severe dementia, the exact same interventions act predominantly by clearing irreversibly senescent, dystrophic cells, thereby terminating chronic senescence-associated secretory phenotype signaling and reducing bystander neuronal injury [42,236]. Consequently, clinical candidate selection should be stratified accordingly: while preventative rejuvenation modalities hold maximal promise in younger, biomarker-positive subjects prior to extensive neurodegeneration, senolytic clearance and cellular replacement strategies offer the greatest clinical benefit in older patients with moderate-to-advanced AD exhibiting established neurodegenerative burdens and elevated neuroinflammatory/senescence biomarker profiles [234,240].

### 5.6. Cell-Based Therapy Modulation of Microglia

Mesenchymal stem cell transplantation—mesenchymal stem cells (MSCs) represent a potent cell-based immunomodulatory strategy for AD, acting primarily through the reprogramming of microglial activation states rather than neuronal replacement. MSCs encompass a heterogeneous family of multipotent stromal cells derived from diverse tissue sources, including bone marrow (BM-MSCs), adipose tissue (AD-MSCs), umbilical cord blood (UCB-MSCs), umbilical cord Wharton’s jelly (WJ-MSCs), placenta (P-MSCs), each has demonstrated the capacity to diminish AD pathology in preclinical models [73,128].

Mechanistically, MSCs are not merely passive cellular fillers but active regulators of the brain’s innate immune environment. Their therapeutic benefits are associated with decreased Aβ accumulation through immunomodulatory suppression of BACE-1 expression, as well as the secretion of trophic factors that reduce neuroinflammation, lower amyloid burden and enhance endogenous neurogenesis [73,241]. This immunomodulation operates through several distinct pathways. First, MSCs secrete stem cell factor, which binds to the c-kit receptor upregulated on activated microglia following brain injury [242], driving polarization toward a protective phenotype [243]. Second, MSC-mediated microglial modulation involves the prevention of MAPK phosphorylation, thereby reducing the transcription of inflammation-associated genes [244]. Third, MSCs promote polarization toward a neuroprotective state via TGF-β and C-X3-C motif chemokine ligand 1 (CX3CL1) signaling and IL-10 secretion further suppresses iNOS expression through inhibition of NF-κB activity in microglia [227].

The effects of MSC-mediated microglial reprogramming are evident at both molecular and behavioral levels. In APP/PS1 transgenic mouse model, AD-MSC transplantation improved spatial learning and memory while promoting the accumulation of alternatively activated microglia around and within Aβ plaques. These changes were accompanied by reduced Aβ deposition, lower BACE-1 expression, and decreased tau hyperphosphorylation, likely through immunomodulatory suppression of BACE-1 and enhanced microglial production of Aβ-degrading enzymes such as neprilysin [245]. In the 5xFAD mouse model, UCB-MSCs attenuated tau hyperphosphorylation through galectin-3 (GAL-3) secretion [246], while WJ-MSCs reduced Aβ burden and increased anti-inflammatory cytokine expression following intravenous administration [247]. AD-MSCs promoted endogenous neurogenesis in the subgranular and subventricular zones and reduced oxidative stress in the APP/PS1 mouse model [128]. Amniotic MSC transplantation improved cognition and synaptic density via BDNF-mediated hippocampal neurogenesis [248].

Beyond cell transplantation, MSC-derived extracellular vesicles (MSC-EVs) have emerged as a cell-free alternative that retains the immunomodulatory and neuroprotective properties of their parent cells. MSC-EVs carry microRNAs, notably miR-29c-3p, that inhibit BACE1 expression and activate the Wnt/β-catenin pathway, while also restoring autophagy function and accelerating the clearance of abnormal protein aggregates. Non-invasive intranasal administration of MSC-EVs in experimental AD mice has been shown to increase neuronal density and reduce hippocampal shrinkage [249]. Preclinical evidence also suggests that preconditioning MSCs, for instance, by prior exposure to an AD environment or incubation with dimethyl fumarate, enhances their survivability, proliferation, and therapeutic potency, pointing to strategies that may bridge the efficacy gap between rodent studies and human trials [247]. Despite these advances, the long-term consequences of microglial replacement remain uncertain, and donor-derived cells may not fully reproduce the transcriptional and functional properties of endogenous microglia. Moreover, established hematopoietic stem cell transplantation approaches are limited by substantial morbidity and low brain chimerism, highlighting important translational barriers for microglial replacement strategies [250,251].

Translational status and limitations—the translational trajectory of MSC therapy in AD, however, remains cautious. Of the few completed phase I trials, a single stereotactic infusion of human UCB-MSCs into the hippocampus and precuneus of patients with mild-to-moderate AD was deemed feasible, safe, and well-tolerated, with no serious adverse effects over two years of follow-up; yet it failed to replicate the therapeutic effects observed in animal models [132]. A phase I/IIa trial of three repeated intraventricular administrations of NEUROSTEM^®^ (human UCB-MSCs) via an Ommaya reservoir completed evaluation in 2019, but failed to demonstrate significant slowing of clinical disease progression, while underscoring the procedural morbidity and surgical risks of chronic ventricular access devices in elderly patients [252,253]. In response to these procedural limitations, clinical evaluation has shifted toward peripheral, non-invasive delivery modalities. Multiple intravenous trials are exploring autologous adipose-derived MSCs (HB-adMSCs/AstroStem; NCT03117738) [254]. Furthermore, in a randomized, double-blind Phase IIa trial (CLEAR MIND; NCT05233774), intravenous administration of allogeneic bone marrow-derived MSCs (laromestrocel/Lomecel-B™) met its primary safety endpoint, with treated mild AD patients exhibiting a significant slowing in whole-brain and hippocampal volume loss on MRI alongside preliminary stabilization on composite cognitive assessments and reductions in neuroinflammatory diffusion tensor imaging metrics [129,130,131]. Notably, a Phase I/II trial (NCT04388982) of intranasal allogeneic adipose MSC-derived exosomes has begun evaluating this cell-free approach in AD [133,249]. Despite these developments, fundamental translational barriers persist. Intravenously administered MSCs rapidly become trapped in the pulmonary capillary bed, resulting in exceptionally low CNS homing and requiring therapeutic benefit to be mediated almost entirely by transient peripheral paracrine signalling [255]. For cells that do reach or are directly delivered to the CNS, the chronically inflamed, proteotoxically hostile microenvironment of the advanced human AD brain limits long-term graft survival and risks inducing donor cell senescence or phenotypic silencing [256]. Preconditioning approaches, such as priming MSCs with inflammatory cues or small-molecule activators prior to infusion, have shown preclinical potential to enhance survivability and microglial reprogramming potency [257]. However, dedicated, adequately powered Phase III trials remain necessary to confirm whether peripheral secretome signaling or cell-free vesicle platforms can elicit sustained central microglial remodeling and translate structural neuroprotection into long-term clinical preservation. The therapeutic benefit of cell therapies depends on disease timing: during early mild cognitive impairment, MSC secretomes stimulate microglial clearance and halt amyloid deposition [73,245], whereas in late-stage dementia, the exact same paracrine factors act primarily by reducing chronic neuroinflammation and cytokine toxicity, as shown in preclinical research [244,258,259]. Consequently, while preclinical animal models demonstrate robust microglial phagocytic reprogramming and aggregate clearance following MSC administration, these mechanisms have not translated into proven clinical disease modification in humans. Early invasive delivery approaches (e.g., stereotactic or intraventricular NEUROSTEM^®^ infusions) failed to arrest clinical progression [133,254], and recent exploratory Phase IIa intravenous trials (such as laromestrocel/Lomecel-B™) provide preliminary volumetric and safety signals that require rigorous confirmation in adequately powered Phase III clinical trials [132,253,260].

Manufacturing, immunological, and regulatory challenges in cell-based therapies: When it comes to treatment consistency, donor-to-donor variability remains a fundamental hurdle for both autologous and allogeneic MSC platforms. Cellular potency, secretome composition, and immunomodulatory capacity fluctuate substantially depending on donor age, tissue source, and medical comorbidities. Furthermore, cell senescence induced by extensive ex vivo expansion alters microRNA and trophic factor packaging, leading to inconsistent paracrine signaling and variable microglial reprogramming across manufacturing batches [261]. Also, industrial-scale biomanufacturing of live cellular products requires tightly controlled bioreactor systems [262]. Unlike stable chemical formulations, live cells are vulnerable to cryopreservation injury, shear stress, and viability loss during thawing and formulation, which compromises final post-thaw potency. Establishing validated potency assays that directly correlate in vitro phenotypic markers with in vivo central neuroimmune modulation remains a major Chemistry, Manufacturing, and Controls milestone [263]. Immune responses although MSCs are traditionally characterized as “immune-evasive” due to low baseline MHC-I and absent MHC-II expression, repeated administration of allogeneic cell products can trigger host allo-recognition. In vivo exposure to an inflammatory, IFN-γ-rich microenvironment upregulates surface MHC-I and MHC-II on transplanted MSCs, eliciting donor-specific antibody generation, complement activation, and accelerated immune clearance that preclude repeated dosing [260].

Advanced Therapy Medicinal Products (ATMPs) face rigorous scrutiny from regulatory agencies (such as the FDA and EMA). Mandates include strict verification of genomic stability to exclude insertional transformation, karyotypic abnormalities, or spontaneous tumorigenesis during extended in vitro passaging. Moreover, regulatory frameworks enforce multi-year long-term monitoring protocols (typically 5 years for non-genetically modified cell products) to surveil potential ectopic tissue formation, chronic peripheral immune sensitization, and delayed neuroinflammatory exacerbations [258,264].

### 5.7. Gene-Based, Delivery Strategies and Engineered Cellular Approaches

#### 5.7.1. Gene Therapy Targeting Microglial Pathways and Engineered Cellular Strategies

Accumulating evidence from human genetics, transcriptomics and functional studies has established TREM2 and APOE as key regulators of microglial responses in AD and among the most significant genetic determinants of disease risk. These pathways orchestrate the transition of microglia from a homeostatic state to disease-associated phenotypes. Given their central role in shaping microglial function and influencing amyloid and tau pathology, TREM2 and APOE have emerged as targets for disease-modifying interventions. This genetic and mechanistic rationale has fuelled the development of therapeutic strategies ranging from small-molecule modulation to direct genetic intervention like gene-silencing and gene-editing approaches designed to restore or reprogram protective microglial functions [259].

#### 5.7.2. Direct Gene Therapy for TREM2

Preclinical gene-delivery strategies aimed at increasing TREM2 expression, including lentiviral-mediated transfer and transgenic overexpression, have demonstrated therapeutic effects in AD rodent models by reducing amyloid burden, limiting tau pathology, and improving cognitive outcomes [149,150]. Although TREM2 gene therapy has shown therapeutic potential, its clinical application is limited by concerns regarding toxicity, immunogenicity, and inflammatory side effects. Emerging microglia-targeted detachable albumin nanocarrier and biomimetic nanovesicle platforms have been designed to improve delivery efficiency, enhance microglial phagocytosis, and simultaneously modulate complementary pathways, such as TREM2 upregulation and BACE1 suppression, to maximize therapeutic efficacy [118]. Nanocarrier-based approaches for microglial modulation in AD remain at a preclinical stage, with substantial differences in the strength of evidence supporting BBB penetration and cellular targeting. Among the available platforms, an albumin-shelled, NCAM-targeted polyplex delivering a TREM2-encoding plasmid provided quantitative evidence of brain exposure, reaching 2.17% of the injected dose per gram of brain, together with measurable plasma pharmacokinetics and reduced accumulation in the liver, spleen and kidneys compared with the uncoated carrier [265]. However, microglial localization reached 41.9%, supporting preferential targeting rather than true microglial specificity [265]. By comparison, a dual-targeted liposomal system co-delivering a TREM2 antisense oligonucleotide and resveratrol demonstrated BBB transport in vitro and increased brain localization by fluorescence imaging in APP/PS1 mice, but quantitative brain exposure and plasma pharmacokinetics were not reported [266]. Other scavenger-receptor-targeted nanoparticles have so far been evaluated only in cellular models, without assessment of BBB penetration in vivo [267]. These differences are important because brain-associated fluorescence or nanoparticle uptake by phagocytic microglia does not necessarily demonstrate effective BBB transcytosis or selective microglial targeting [268,269]. Moreover, although short-term toxicity and physicochemical properties have been evaluated for some platforms, immunogenicity, long-term clearance and accumulation, systemic biodistribution, and manufacturing reproducibility and scalability remain important translational considerations that require more systematic evaluation [268]. Thus, current evidence supports the feasibility of microglia-oriented nano-delivery in experimental models, but clinical applicability and selective microglial targeting in humans remain unestablished.

#### 5.7.3. APOE-Directed Gene Therapy and Gene Editing

Gene-editing strategies that convert APOE4 to the lower-risk APOE3 or APOE2 isoforms have shown therapeutic potential in AD. Zinc-finger nucleases have been used to convert APOE4 to APOE3, in human in vitro models, which reduced Aβ production and accumulation while improving glial amyloid clearance, supporting APOE editing as a promising disease-modifying approach [151,153]. Recent studies conducted by Günaydin and collaborators have employed prime-editing technology delivered through dual adeno-associated virus (AAV) vectors to achieve in vivo APOE4-to-APOE3 conversion in humanized APOE4 knock-in mice. Following optimization of guide RNAs and AAV-based delivery systems, efficient editing was achieved in both the liver and brain, including widespread editing after intrahippocampal, cisterna magna, or systemic administration [152].

#### 5.7.4. iPSC-Derived Microglia

Recent advances in the generation of induced microglia-like cells (iMGs) from human induced pluripotent stem cells (hiPSCs) have provided a powerful platform for studying human microglial function and disease-relevant phenotypes in a controlled in vitro environment [85]. In vitro iMGs can be modified by CRISPR/Cas9 to generate isogenic lines carrying or correcting AD risk alleles like TREM2-R47H or APOE4, enabling direct functional comparisons on a uniform genetic background [270]. It has been demonstrated that xenotransplantation of hiPSC-derived hematopoietic progenitors into neonatal human CSF1-expressing immunodeficient mice gives rise to chimeric microglia that exhibit mature, ramified morphology, restored homeostatic signatures and transcriptional profiles closely resembling those of primary human microglia [271]. While these chimeric platforms provide unprecedented preclinical utility for dissecting human myeloid drug responses in vivo, their application remains investigative rather than an immediate autologous replacement therapy, given the severe immunological barriers, graft survival constraints, and conditioning toxicities inherent to human cell engraftment.

Translational status and limitations—translating gene-delivery, gene-editing, and engineered cellular strategies into clinical therapeutics remains constrained by specific safety, delivery, and host-environment barriers. Although preclinical viral augmentation of TREM2 demonstrates neuroprotection, its clinical application is limited by toxicity, immunogenicity, and inflammatory side effects, spurring the development of detachable albumin and biomimetic nanovesicles to overcome these delivery hurdles. In human clinical trials, therapeutic modulation of the APOE landscape has advanced through central gene augmentation rather than direct genomic cleavage. The first-in-human Phase I/II clinical trial (LX1001; NCT03634007) in APOE4 homozygous patients ranging from mild cognitive impairment to mild or moderate dementia due to AD, evaluated intracisternal administration of an AAVrh.10 vector encoding protective human APOE2 into the cerebrospinal fluid of symptomatic APOE4/4 homozygotes. Preliminary clinical readouts confirmed safe intracisternal delivery, sustained expression of APOE2 protein in human CSF, and favorable biomarker trends, providing clinical validation for gene-based conversion of the high-risk CNS lipid microenvironment [125]. However, this approach represents early-phase safety validation of passive protein expression rather than definitive proof of cognitive stabilization or broad genomic conversion in human cohorts. Additional development candidates aim to enhance this research, including LX1021, which expresses the rare Christchurch-variant APOE2, and LX1020, which couples APOE2 delivery with concurrent microRNA-mediated suppression of pathogenic APOE4 [272]. hiPSC-derived microglia and chimeric xenotransplantation models remain investigative platforms rather than viable autologous replacement therapies due to severe immunological barriers, poor graft survival, and the toxicities inherent to host conditioning regimens [273].

Gene-based strategies confer clinical protection through distinct stage-dependent mechanisms: in early mild cognitive impairment, central expression of protective APOE2 primarily counteracts the local lipid and metabolic deficits imposed by APOE4, restoring microglial surveillance and halting nascent plaque consolidation [74,124,125], whereas in advanced dementia, the same vector-derived protein helps by neutralizing extracellular APOE4 neurotoxicity and dampening secondary neuroinflammatory cascades to protect surviving synapses within an already altered CNS microenvironment [75]. Accordingly, clinical utility is largely restricted to genetically enriched cohorts: symptomatic APOE4 homozygotes in early dementia stages with preserved ventricular and cortical anatomy represent the subgroup most likely to achieve lasting phenotypic conversion and disease modification, whereas unselected or severely atrophic dementia populations derive negligible structural recovery [152,274].

#### 5.7.5. Nanoparticle Delivery Systems Targeting Microglia

In preclinical studies, engineered nanocarriers have demonstrated substantial utility in overcoming BBB restrictions and enhancing drug bioavailability within the central nervous system. Diverse nanoplatforms, including liposomes, polymeric nanoparticles, solid lipid nanoparticles, and dendrimers, have been developed to enhance drug solubility, stability, bioavailability, and CNS delivery efficiency [275]. Among these, poly(D,L-lactic-co-glycolic acid) (PLGA)-based nanoparticles have shown promise for microglia-directed therapies. For example, PLGA-mediated delivery of p16^ink4a^ siRNA enhanced microglial Aβ phagocytosis, reduced amyloid plaque burden, decreased the accumulation of senescent microglia, and improved cognitive performance in AD mice [156]. Another PLGA-based therapeutic strategy employed an engineered biomimetic nanoplatform (Gas6-NV-NPs) in which PLGA nanoparticles co-encapsulating rapamycin and nicotinamide riboside were coated with membranes derived from Gas6-overexpressing neural stem cells that in 5xFAD mice model demonstrated efficient brain targeting, reduced amyloid burden, and facilitated neuronal repair [155]. Beyond polymeric nanoparticles, biomimetic nanovesicle systems have emerged as highly versatile delivery vehicles. Recent advances in preclinical models, employ microglia-derived nanovesicles (AR@ENV) generated through the Microglia–Liposome Fusion Extrusion (MiLi-FE) platform and designed to simultaneously activate chaperone-mediated autophagy (CMA) and macroautophagy. AR@ENV co-delivers AR7, a CMA inducer that antagonizes retinoic acid receptor-α (RARα), and rapamycin, an mTOR inhibitor that stimulates macroautophagy. Owing to their microglial membrane origin, these nanovesicles cross the BBB, in preclinical mouse models, accumulate at neuroinflammatory sites, and are internalized by neurons. In AD mouse models, synchronized activation of both autophagic pathways enhanced the clearance of Aβ and other toxic protein aggregates, restored proteostasis, reduced neuroinflammation, and significantly improved cognitive performance [276].

Translational status and limitations—translating microglia-targeted nanocarriers to human therapeutics faces critical pharmacological and biomanufacturing roadblocks. Scalable, reproducible synthesis of biomimetic membrane coatings and extruded vesicles under GMP standards remains technically challenging regarding batch uniformity and long-term stability. Systemically, rapid clearance by the reticuloendothelial system restricts human BBB penetration far more strictly than in rodents. Furthermore, the long-term safety of repetitive carrier dosing in elderly dementia patients, including risks of chronic nanomaterial accumulation, carrier-induced lysosomal overload, and adaptive immune responses to cell-membrane coatings, remains unproven in clinical trials [277,278,279,280]. The clinical utility of targeted nanoparticles depends entirely on the timing of intervention. In early mild cognitive impairment, functionalized nanoparticles can boost microglial phagocytosis and amyloid compaction, providing the greatest benefit to early-stage APOE ε4 or TREM2 risk carriers. In late-stage dementia, however, exhausted microglia can no longer clear aggregates effectively; here, the same delivery systems should focus on suppressing harmful inflammatory signaling and preserving remaining neural circuits. Selecting patients based on disease stage, genotype, and neuroinflammatory markers is therefore essential to achieve meaningful efficacy while avoiding off-target toxicity [281].

Manufacturing, immunological, and regulatory challenges in gene-based therapies—given the progressive and chronic course of neurodegenerative diseases, sustained transgene expression is crucial for maintaining long-term therapeutic efficacy. Durable expression may reduce the need for repeated administration, thereby limiting immune responses, treatment-related toxicity, and associated costs. Current strategies to improve long-term safety and efficacy include the development of less immunogenic viral vectors and the use of inducible or regulatable expression systems [282,283,284]. The clinical translation of gene therapies is associated with substantial regulatory challenges, as demonstrating their safety and efficacy requires rigorous clinical evaluation. The prolonged persistence of viral vector genomes and the irreversible nature of genome-editing interventions necessitate rigorous long-term safety surveillance. FDA and EMA regulatory frameworks may require extended follow-up of patients receiving certain gene therapies, with monitoring periods reaching up to 15 years depending on the vector, intervention, and associated risks. Long-term surveillance focuses on delayed adverse events, including insertional oncogenesis, persistent or ectopic transgene expression, vector-related complications, and late-onset neurological toxicity. In elderly and potentially frail AD populations, such prolonged follow-up presents additional challenges related to patient retention, sustained funding, and longitudinal safety assessment [258,285]. High-dose gene delivery to the CNS may be associated with important organ-specific toxicities. Intrathecal or systemic administration of AAV vectors has been linked to dorsal root ganglion injury, including inflammatory cell infiltration, sensory neuron degeneration, and secondary axonal damage. Systemic vector exposure may also contribute to hepatotoxicity and complement-mediated thrombotic microangiopathy. Genome-editing approaches introduce additional risks, including off-target DNA cleavage, chromosomal rearrangements, and prolonged Cas9 expression, which may promote sustained innate immune signaling and cellular stress or senescence [284]. Achieving uniform therapeutic expression across the human brain remains a key pharmacological challenge for AD gene therapies. Viral vectors administered via the cerebrospinal fluid rely on passive diffusion and glymphatic clearance, creating steep concentration gradients that result in supraphysiological transgene levels near delivery sites but suboptimal exposure in vulnerable deep structures such as the hippocampus [282]. Furthermore, standard rAAV serotypes selectively transduce neurons and astrocytes with minimal native tropism for microglia, necessitating reliance on indirect paracrine cross-correction (e.g., secreted APOE2 or sTREM2) that varies widely across patients due to individual atrophy and CSF flow dynamics. In somatic gene-editing platforms, incomplete vector distribution inevitably produces cellular mosaicism, leaving it clinically unresolved whether partial allelic conversion provides an adequate phenotypic threshold to halt neurodegeneration [283]. When it comes to scalability, transitioning CNS gene therapies from ultra-rare pediatric conditions to highly prevalent populations like AD create unprecedented Chemistry, Manufacturing, and Controls constraints. Producing industrial-scale, high-titer recombinant viral vectors require large-volume suspension bioreactors that suffer from substantial batch-to-batch variability and prohibitive costs. A major technical bottleneck lies in the downstream separation of genome-containing capsids from non-functional or partially packaged capsids. These empty capsids fail to deliver the therapeutic transgene while serving as competitive antigenic decoys that trigger host inflammatory cascades, directly complicating dose standardization and global commercial viability [262,283].

### 5.8. Cannabinoid and Endocannabinoid Modulation of Microglial Cells in AD

Microglia possess a fully functional endocannabinoid system (ECS), comprising cannabinoid receptors (CB1 and CB2), endogenous cannabinoids (endocannabinoids; eCBs) such as anandamide (AEA) and 2-arachidonoylglycerol (2-AG), as well as the enzymatic machinery required for their synthesis and degradation [286,287]. Beyond endocannabinoids, the cannabinoid family also includes synthetic cannabinoids, which are laboratory-engineered compounds designed to mimic or modulate cannabinoid receptor signaling, and phytocannabinoids, naturally occurring bioactive molecules derived from the *Cannabis sativa* plant, including Δ9-tetrahydrocannabinol (THC) and cannabidiol (CBD) [288,289,290]. Activation of microglial cannabinoid receptors modulates multiple intracellular signaling pathways involved in inflammatory responses [286]. In particular, endocannabinoids suppress MAPK signaling and regulate extracellular signal-regulated kinase, c-Jun N-terminal kinase (JNK), and p38 phosphorylation, resulting in increased production of anti-inflammatory mediators such as IL-10 and reduced release of pro-inflammatory cytokines [291,292]. Both AEA and 2-AG also inhibit the expression of inflammatory mediators including IL-15, thereby contributing to the resolution of neuroinflammation [292]. Under neuroinflammatory conditions, microglia constitute a major source of eCB production within the CNS, generating substantially higher levels of these lipid mediators than neurons or other glial populations [293,294]. Notably, endocannabinoid metabolism is closely linked to microglial activation states. Polarization toward anti-inflammatory, resolving phenotypes is associated with the upregulation of eCB-synthesizing enzymes, including diacylglycerol lipase alpha (DAGLα) and N-acylphosphatidylethanolamine phospholipase D (NAPE-PLD), alongside reduced expression of degradative enzymes, thereby promoting increased endocannabinoid availability and signaling [295]. In human post-mortem AD brain tissue, CB2 is upregulated in plaque-associated microglia, as a compensatory response to Aβ-induced inflammation and cytokine exposure, and absent in resting microglia [292,296]. Activation of CB2 receptors promotes microglial proliferation, migration, and phagocytic activity while suppressing the production of TNF-α, reactive oxygen species, and other inflammatory mediators. Consequently, CB2 signaling is considered a key regulator of the anti-inflammatory and neuroprotective functions of microglia. In contrast, CB1 receptors are constitutively expressed at relatively low levels and remain largely unaffected by changes in microglial activation status [292,294]. This suggests that CB2 functions as an inducible receptor that is expressed in response to inflammatory stimuli, a characteristic shared by cells of the macrophage lineage. Importantly, the elevated CB2 expression observed in responsive and primed microglia indicates the existence of a functionally relevant therapeutic window during which these cells may be particularly sensitive to cannabinoid-mediated modulation [288,293]. Chronic administration of the selective CB2 agonist JWH-133 in pre-symptomatic and early symptomatic APPSwe/PS1ΔE9 mice model significantly improved cognitive performance while reducing plaque-associated microgliosis and the expression of IL-1β, IL-6, TNF-α, and interferon-gamma (IFN-γ). These anti-inflammatory effects were accompanied by reduced oxidative stress and decreased tau hyperphosphorylation [158]. Similar findings were reported in the Tg2576 mouse model, where JWH-133 reduced TNF-α and COX-2 expression and improved memory deficits. In contrast, chronic treatment with WIN55, 212-2 failed to produce comparable effects on microglial density, whereas JWH-133 significantly decreased the number of reactive microglia [297]. Other CB2 agonists, including HU-308 and AM1241, have demonstrated comparable anti-inflammatory and neuroprotective effects [298], with AM1241 additionally enhancing mitochondrial biogenesis through peroxisome proliferator-activated receptor γ coactivator 1-α (PGC-1α)-dependent pathways [299]. The natural CB2 agonist β-caryophyllene likewise promotes polarization of microglia toward an anti-inflammatory phenotype, characterized by increased production of IL-10, ARG1, urea, and the antioxidant glutathione, while simultaneously reducing the expression of pro-inflammatory mediators, including IL-1β, TNF-α, prostaglandin E2 (PGE2), iNOS, nitric oxide, and ROS [300]. Pharmacological modulation of endocannabinoid metabolism has also shown therapeutic potential. Inhibition of MAGL reduces Aβ-induced toxicity and neuroinflammation through suppression of extracellular signal-regulated kinases 1 and 2 (ERK1/2), NF-κB, and COX-2 signaling, while fatty acid amide hydrolase (FAAH) deficiency promotes disease-associated microglial phenotypes characterized by increased expression of phagocytic markers such as TREM2 and cathepsin D, resulting in enhanced Aβ clearance and reduced plaque burden. These findings suggest that enhancing endogenous cannabinoid signaling may be an effective strategy for restoring microglial homeostasis and limiting AD progression [292,301]. The potential synergistic effects of THC and CBD have been investigated in experimental models of AD. In a 6-month-old APP/PS1 mouse model, intraperitoneal administration of THC, CBD, or their combination for five weeks improved performance in learning and memory tasks, including novel object recognition and active avoidance tests. However, only the combined THC/CBD treatment significantly reduced soluble Aβ42 levels and cortical gliosis, indicating a synergistic effect on AD-related neuropathology. Notably, when the same treatment paradigm was applied to 12-month-old APP/PS1 mouse model, these reductions in Aβ42 burden and gliosis were no longer observed, suggesting that cannabinoid-mediated neuroprotective mechanisms may be more effective during the early stages of disease progression [302,303].

Translational status and limitations: In human clinical trials, the therapeutic evaluation of cannabinoids in AD has diverged substantially from preclinical disease-modifying hypotheses. To date, clinical investigations have focused almost exclusively on managing neuropsychiatric and behavioral symptoms of dementia, such as agitation, nocturnal motor activity, and anorexia in moderate-to-severe AD cohorts [304,305]. Randomized, placebo-controlled trials assessing synthetic THC analogues, including nabilone and dronabinol, demonstrated modest reductions in agitation scales but reported zero cognitive enhancement or biomarker alteration, while presenting risks of dose-dependent sedation and transient lethargy in frail elderly individuals [304,306]. While selective CB2 receptor agonists demonstrate compelling microglial reprogramming in rodent paradigms, no selective CB2 agonist has successfully advanced through definitive Phase II/III clinical testing for cognitive endpoints in human AD, leaving the disease-modifying potential of cannabinoid-directed microglial modulation unvalidated in clinical practice. Current clinical evidence is restricted entirely to synthetic cannabinoid analogues (e.g., nabilone, dronabinol) utilized off-label for the palliative, symptomatic management of refractory neuropsychiatric symptoms and agitation in advanced dementia, demonstrating no measurable impact on amyloid pathology, tau spreading, or cognitive decline. Systemic translation must navigate significant pharmacological hurdles, including receptor desensitization following chronic CB2 agonist exposure, narrow therapeutic windows, variable central bioavailability, and the psychoactive or sedative liabilities of CB1 cross-reactivity in elderly, cognitively frail patients [307]. Cannabinoid-based therapies operate through fundamentally different mechanisms depending on disease timing. Early in the disease course, CB2 agonists preserve baseline surveillance and promote non-inflammatory aggregate clearance. In advanced stages, where microglia are chronically primed and amyloid clearance is impaired, identical receptor stimulation acts instead to quench neurotoxic cytokine cascades. Although progressive upregulation of CB2 receptors alongside amyloid and tau pathology creates a larger pharmacological target in severe disease, microglial exhaustion limits the potential for tissue repair [295]. As a result, very advanced patients with extensive neuronal loss derive little structural benefit, whereas early presymptomatic individuals lack an activated substrate. Clinical efficacy will therefore rely on enrolling patients within a defined intermediate window, specifically those with early-to-moderate impairment and biomarker evidence of active neuroinflammation.

## 6. On-Target Versus Off-Target and Multi-Cellular Effects of Microglia-Directed Therapies

A major challenge in the development of microglia-targeted therapies arises from the extensive bidirectional communication between microglia and other cellular components of the brain microenvironment, including astrocytes, neurons, oligodendrocytes, endothelial cells, and peripherally derived immune cells. Signaling pathways involving TREM2, complement components, and cytokine networks participate in complex intercellular interactions, such that even highly selective interventions targeting microglia may elicit downstream effects beyond the intended cellular population [84,308]. For instance, alterations in microglial cytokine secretion and complement signaling may influence astrocytic activation states and disrupt the regulation of synaptic elimination, potentially compromising neuronal connectivity [72,217]. Similarly, changes in microglial inflammatory activity may affect blood–brain barrier integrity and neurovascular function, while therapeutic agents acting on targets also expressed by peripheral immune cells may produce unintended systemic immunological effects [145,260]. These interconnected responses highlight the need to evaluate microglia-directed interventions not merely in terms of their cell-specific effects, but also in relation to their broader consequences for neuronal, glial, vascular, and peripheral immune homeostasis (Table 2).

## 7. Limitations and Future Perspectives

A primary limitation of the literature reviewed herein is the heavy reliance on transgenic rodent models driven by familial amyloid mutations (such as 5xFAD, APP/PS1, or Tg2576). While these models are instrumental for interrogating acute amyloid clearance and plaque-microglia interactions, they fail to recapitulate the multifactorial etiology, prolonged chronological aging, and spontaneous tau neurofibrillary tangle spreading characteristic of sporadic, late-onset human AD. Furthermore, single-nucleus transcriptomic profiling underscores that murine DAM signatures do not fully mirror the diverse, stochastic microglial states identified in post-mortem human brains. Interspecies variations in myeloid turnover kinetics, spanning days in rodents versus decades in humans, further complicate the clinical translation of strategies such as microglial depletion-repopulation regimens. Beyond model-specific constraints, clinical translation is strictly governed by disease stage and host-intrinsic heterogeneity. First, the beneficial or detrimental effects of microglial responses are strongly influenced by disease stage, suggesting that the timing of therapeutic intervention represents a critical determinant of treatment efficacy. Second, substantial cellular and interindividual heterogeneity exists, influenced by aging, sex, genetic background, environmental factors and systemic comorbidities, which fundamentally regulates microglial reactivity and dictates clinical treatment response. Chronological aging promotes microglial dystrophy, autophagic decline, intracellular lipid-droplet accumulation, and process retraction. Consequently, pro-clearance and metabolic interventions (e.g., PPAR agonists or myeloid growth factors) that succeed in juvenile animal models often underperform in elderly cohorts whose microglia have reached an uncoupled, senescent state dominated by senescence-associated secretory phenotype signaling [39,42]. Microglial transcriptomic trajectories exhibit pronounced sexual dimorphism. Under amyloid stress, female microglia undergo an accelerated transition toward DAM and IRM phenotypes, driving more sustained pro-inflammatory signaling and heightened microvascular vulnerability compared to males [312,313]. Clinical trials must incorporate sex-stratified analysis, as immunomodulatory, hormonal, and lipid-targeting interventions may yield divergent efficacy between sexes. The APOE ε4 allele impairs microglial motility, impedes ABCA1-mediated lipid recycling, and blunts non-phlogistic aggregate clearance compared to neutral ε3 or protective ε2 isoforms. Homozygous ε4\ε4 patients exhibit an intrinsically primed, pro-inflammatory microglial baseline that accelerates plaque seeding and secondary tau dissemination. While ε4 carriers represent the primary candidates for lipid-reprogramming therapies (e.g., obicetrapib) and viral APOE2 gene augmentation, their elevated neurovascular fragility requires cautious dosing regimens [50,74,224]. Concomitant vascular pathology, such as cerebral amyloid angiopathy, microvascular rarefaction, and endothelial tight-junction disruption, alters perivascular microglial surveillance. In patients with substantial vascular pathology, rapid microglial mobilization or aggressive aggregate dissolution markedly increases the risk of microhemorrhages and vasogenic edema, emphasizing the necessity of pre-treatment vascular neuroimaging [217,314]. Peripheral insulin resistance, type 2 diabetes mellitus, and systemic dyslipidemia sustain chronic low-grade systemic inflammation that primes central myeloid cells across the BBB. Microglia in diabetic or dyslipidemic hosts exhibit mitochondrial stress, impaired TREM2–SYK signaling, and defective lysosomal degradation, while altered endothelial transporter function can compromise the central bioavailability and pharmacokinetics of administered small molecules and nanocarriers [315,316,317]. Third, the identification and validation of reliable biomarkers capable of monitoring microglial activation states in vivo remain essential for assessment of therapeutic response. Future research should focus on the precise characterization of glial phenotypes across different stages of disease progression, the identification of biomarkers that accurately reflect functional glial states, and the integration of multi-omics approaches with advanced neuroimaging technologies. In parallel, emerging technologies such as gene therapy, microglial replacement strategies, including induced pluripotent stem cell-based approaches, and targeted nanomedicine platforms offer new opportunities to modulate microglial function with greater specificity and precision. Finally, the most effective therapeutic strategies will likely combine microglia-targeted interventions with established anti-amyloid, anti-tau, and symptomatic treatments. Such multimodal approaches may restore neuroimmune homeostasis while simultaneously targeting the multiple pathological processes that drive AD progression. As our understanding of microglial biology continues to advance, these cells are poised to become central therapeutic targets in the development of next-generation disease-modifying therapies for AD.

## 8. Conclusions

AD is increasingly recognized as a complex neuroimmune disorder in which microglia actively participate in the initiation, propagation, and progression of pathology rather than acting as passive responders to neuronal injury. Evidence accumulated from experimental models, human genetics, transcriptomics, and clinical studies demonstrates that microglia exert highly context-dependent effects throughout disease progression. While microglial responses in the early stages of the disease may exert neuroprotective effects through amyloid clearance, support of tissue repair processes, and maintenance of neuronal homeostasis, chronic microglial activation promotes neuroinflammation, synaptic dysfunction, tau pathology propagation, and neurodegeneration. The growing understanding of microglial heterogeneity has fundamentally reshaped the therapeutic perspective of AD. Rather than classifying microglia into traditional binary phenotypes, current evidence supports the existence of dynamic and functional microglial states that evolve throughout disease progression. Consequently, therapeutic strategies are increasingly shifting away from nonspecific anti-inflammatory approaches toward the selective enhancement of neuroprotective functions, suppression of maladaptive inflammatory responses, reprogramming of glial phenotypes, and modulation of interactions between microglia and their surrounding microenvironment. Rather than treating all interventions equally, therapeutic development must prioritize targets with strong genetic validation, cellular specificity, and disease-stage appropriateness. First, the TREM2-APOE axis is the highest-priority target for early intervention. Agonistic TREM2 activation and APOE lipid reprogramming enhance plaque compaction and lipid homeostasis, offering maximal benefit during prodromal stages, especially in APOE ε4 carriers. Future work must refine CNS-penetrant modulators that avoid accelerating late-stage tau spread. Second, NLRP3 inflammasome blockade serves as the primary strategy for intermediate-to-advanced stages. Inhibiting the NLRP3-caspase-1 cascade disrupts ASC speck cross-seeding and arrests cytokine-driven tau propagation. Developing BBB-permeable compounds that avoid systemic toxicity remains a critical future priority. Third, receptor-targeted nanomedicine and gene-delivery platforms represent the most promising engineering strategies to resolve cellular specificity. These platforms deliver therapeutic cargoes directly to microglia, safely circumventing severe off-target peripheral toxicities. Achieving true disease modification across the Alzheimer’s continuum requires moving beyond monotherapies. The most effective clinical paradigms will integrate these prioritized microglial modulators into multimodal regimens, guided by patient stratification accounting for age, sex, APOE genotype, and metabolic comorbidities. Finally, future therapeutic strategies may combine microglia-targeted interventions with approved disease-modifying therapies, investigational tau-directed approaches, and established symptomatic treatments. Such multimodal strategies could simultaneously address protein aggregation, neuroimmune dysfunction, and clinical symptoms; however, their potential additive benefit and safety require validation in appropriately designed clinical studies. As our understanding of microglial biology continues to advance, these cells are poised to become central therapeutic targets in the development of next-generation disease-modifying therapies for AD.

## Figures and Tables

**Table 1:** Main therapeutic strategies targeting microglia in AD. Upward (↑) and downward (↓) arrows indicate increased or decreased expression or activity of the indicated molecular pathways or biological processes, respectively.

Development Stage	Target/Compound	Mechanism	References
Clinical Development	TREM2 agonistic antibody—AL002 [Phase II completed] Small-molecule TREM2 agonist- VG-3927 [Phase I completed]	↑ TREM2–DAP12–SYK signaling and phagocytosis	[118,119,120,121,122,123]
	APOE-directed therapies—CS-6253 [Phase I completed]	Modulation of APOE-dependent lipid metabolism and microglial phenotype	[124,125,126,127]
	Mesenchymal stem cells (MSCs) and MSC-derived exosomes—Lomecel-B, AstroStem [Phase IIa completed], AD-MSC exosomes [Phase I/II]	Immunomodulation of microglial states through secretion of stem cell factor	[128,129,130,131,132,133]
Completed clinical trials	NSAIDs	COX inhibition; ↓ microglial activation	[134,135]
	Minocycline	↓ Microglial activation and pro-inflammatory cytokines	[136,137,138,139]
	PPAR agonists	PPAR activation; ↓ NF-κB signaling	[140,141]
Preclinical stage	Direct NLRP3 inflammasome inhibitors	NLRP3 inflammasome inhibition; ↑ protective microglial phenotype	[78,142,143,144]
	Pharmacological CSF1R inhibition	Transient depletion of resident microglia followed by microglial repopulation	[100,145,146]
	Microglial replacement and senescence targeting	Replacement/modulation of senescent microglia	[147,148]
	TREM2-directed gene therapy through lentiviral-mediated transfer and transgenic overexpression	↑ TREM2 expression	[149,150]
	APOE-directed gene therapy/editing	Conversion of APOE4 to the lower-risk APOE3 or APOE2 isoforms	[151,152]
	CRISPR/Cas9-edited iPSC-derived microglia	Correction of AD-associated risk variants	[153]
	Microglia-targeted nanoparticles delivery systems	Targeted drug delivery to microglia	[154,155,156]
	CB2R agonists	↑ Phagocytosis; anti-inflammatory modulation	[157,158,159]
	Phytocannabinoids (e.g., CBD, THC)	Modulation of neuroinflammation and oxidative stress	[160,161]

**Table 2:** On-target microglial mechanisms, central multi-cellular interactions, and peripheral off-target liabilities of microglia-directed therapies in AD.

Therapeutic Class	Primary Microglial Mechanism (On-Target)	CNS Multi-Cellular Effects (Astrocytes, Neurons, Blood Vessels)	Peripheral Immune and Systemic Off-Target Effects	Bibliography
NSAIDs & aspirin	COX-1/2 inhibition; suppression of microglial NF-κB and cytokine release.	Neurons: Modulates neuronal COX-2 excitotoxicity.	High risk of gastrointestinal ulcerations/bleeding, renal impairment, and cardiovascular events from systemic COX inhibition in elderly patients.	[134,135]
		Astrocytes: Reduces reactive astrogliosis.		
		Endothelium: Suppresses vascular endothelial prostanoids.		
Minocycline & PPAR agonists	Inactivates microglial MMPs and inflammatory kinases; PPARγ-mediated anti-inflammatory polarization.	Astrocytes: Enhances astrocytic Aβ internalization.	Systemic immunosuppression; PPARγ-mediated fluid retention, peripheral edema, cardiac failure risk, and hepatotoxicity.	[172,178,183]
		Endothelium: PPARβ/δ enhances VEGF receptor and endothelial nitric oxide (eNOS).		
		Neurons: Direct anti-apoptotic effects (Bcl-2 upregulation).		
NLRP3 Inflammasome & Cytokine blockers	Blocks ASC speck release, caspase-1 activation, and IL-1β/IL-18 maturation	Astrocytes: Prevents microglial-driven neurotoxic astrocytic conversion.	Systemic immunodeficiency/increased vulnerability to opportunistic infections; off-target hepatotoxicity.	[76,77,142,198]
		Neurons: Protects against excitotoxic and tau kinase hyperactivation.		
TREM2 & APOE modulators	Promotes microglial survival, plaque compaction, lipid recycling via ABCA1.	Astrocytes: Modulates astrocyte–microglia lipid exchange.	Cross-reactivity with peripheral monocytes/macrophages; systemic lipid alterations (severe hypertriglyceridemia with RXR agonists).	[56,119,123,259,309]
		Endothelium: Influences cerebral amyloid angiopathy deposition and perivascular drainage.		
CSF1R inhibitors	Direct depletion and renewal of dysfunctional microglia	Neurons: Loss of plaque barrier in early stages; altered spine density.	Depletion of bone marrow monocytes and tissue macrophages; cholestatic hepatotoxicity; neutropenia.	[100,145,146,229]
Cell-Based therapies (MSCs/EVs)	Paracrine microglial polarization, BACE1 reduction, neprilysin upregulation	Neurons: Promotes endogenous neurogenesis and BDNF release.	Pulmonary capillary microembolization/trapping following IV infusion; host allo-recognition and antibody-mediated clearance on repeated dosing.	[133,241,256,310]
		Astrocytes: Attenuates chronic astrogliosis.		
		Endothelium: Promotes angiogenesis.		
Gene therapies & editing	Sustains APOE2/TREM2 expression; rescues microglial autophagic/lipid flux	Neurons & astrocytes: indirect paracrine cross-correction (secreted APOE2/TREM2); risk of Cas9-mediated off-target cleavage in bystander cells	Complement microangiopathy; acute hepatotoxicity.	[150,152,282,283,284]
Nanocarrier systems	Receptor-mediated intracellular cargo delivery (siRNA, enzymes, anti-inflammatories)	Endothelium: Transcytosis receptor saturation and endothelial shear stress.	Reticuloendothelial system clearance (splenic/hepatic sequestration); carrier accumulation and lysosomal overload in liver/kidneys.	[154,155,276,281,311]
		Neurons: Non-specific endocytosis in off-target neural cells.		
Cannabinoid modulators	CB2-mediated microglial motility, non-inflammatory phagocytosis, and cytokine suppression	Neurons: CB1-mediated psychotropic sedation, cognitive blunting, and synaptic modulation.	Modulates peripheral circulating splenocytes and leukocytes; transient cardiovascular/hypotensive fluctuations.	[157,287,288,289,290,298,301,304]
		Astrocytes: Modulates astrocytic glutamate transport.		

## Data Availability

No new data were created or analyzed in this study. Data sharing is not applicable to this article.

## Abbreviations

The following abbreviations are used in this manuscript:

AD	Alzheimer’s disease
APP	amyloid precursor protein
Aβ	amyloid-β
CNS	central nervous system
ROS	reactive oxygen species
IL-1Ra	interleukin-1 receptor antagonist
IL-10	interleukin-10
TGF-β	transforming growth factor beta
TNF-α	tumor necrosis factor α
RNS	reactive nitrogen species
DAM	disease-associated microglia
IL-1β	interleukin-1β
IL-6	interleukin-6
iNOS	inducible nitric oxide synthase
ARG1	arginase 1
YM1	chitinase-3-like protein 3
COX2	cyclooxygenase-2
CD163	cluster of differentiation 163
NFT	neurofibrillary tangle
MGnD	microglial neurodegenerative phenotype
ARM	activated response microglia
MHC-II	major histocompatibility complex class II
DM	dark microglia
TEM	transmission electron microscopy
IRM	Interferon-response microglia
TLR	Toll-like receptors
IDE	insulin-degrading enzyme
NEP	neprilysin
RAGE	receptor for advanced glycation end products
NLRP3	NOD-, LRR- and pyrin domain-containing protein 3
IL-18	interleukin-18
IL-3	interleukin-3
IL-3Rα	interleukin 3 receptor alpha
GSK-3β	glycogen synthase kinase-3 beta
CSF1R	colony stimulating factor 1 receptor
NSAIDs	nonsteroidal anti-inflammatory drugs
CSF	cerebrospinal fluid
BACE1	beta-site amyloid precursor protein cleaving enzyme 1
BBB	blood–brain barrier
MMPs	matrix metalloproteinase
PPARs	Peroxisome proliferator-activated receptors
GFAP	glial fibrillary acidic protein
IL-4	interleukin-4
MAPK	mitogen-activated protein kinase
APOE	Apolipoprotein E
MSCs	mesenchymal stem cells
BM-MSCs	bone marrow mesenchymal stem cells
AD-MSCs	adipose tissue mesenchymal stem cells
UCB-MSCs	umbilical cord blood mesenchymal stem cells
WJ-MSCs	umbilical cord Wharton’s jelly mesenchymal stem cells
P-MSCs	placenta mesenchymal stem cells
iMGs	microglia-like cells
hiPSCs	human induced pluripotent stem cells
eCBs	endogenous cannabinoids/endocannabinoids
ECS	endocannabinoid system
CB1	cannabinoid receptor 1
CB2	cannabinoid receptor 2
THC	Δ9-tetrahydrocannabinol
CBD	cannabidiol
AEA	anandamide
2-AG	2-arachidonoylglycerol
TREM2	triggering receptor expressed on myeloid cells 2
NF-κB	nuclear factor kappa B
ASC	apoptosis-associated speck-like protein containing a CARD
C1q	complement component 1q
C3	complement component 3
C3aR	complement component 3a receptor
DAP12	DNAX-activating protein of 12 kDa
TYROBP	transmembrane immune signaling adaptor TYROBP
SYK	spleen tyrosine kinase
GLP-1	glucagon-like peptide-1
ASO	antisense oligonucleotide
CD36	cluster of differentiation 36
CD14	cluster of differentiation 14
CD47	cluster of differentiation 47
ERK1/2	extracellular signal-regulated kinases 1 and 2
JNK	c-Jun N-terminal kinase
PGE2	prostaglandin E2
FAAH	fatty acid amide hydrolase
MAGL	monoacylglycerol lipase
DGAT1/2	diacylglycerol O-acyltransferases 1 and 2
ABCA1	ATP-binding cassette transporter A1
IL-1α	interleukin-1 alpha
ARIA	amyloid-related imaging abnormalities
RXR	retinoid X receptor
